# Reduced ROS-associated prophage induction in a *lepA* mutant contributes to increased fluoroquinolone persistence in *Salmonella* Typhimurium

**DOI:** 10.1038/s41598-026-47552-0

**Published:** 2026-04-17

**Authors:** Sebastian Braetz, Magdalena Karp, Andreas Nerlich, Marcus Fulde

**Affiliations:** 1 Institute of Microbiology and Epizootics, School of Veterinary Medicine, Freie Universität Berlin, Robert- von-Ostertag-Str. 8, 14163 Berlin, Germany; 2 Veterinary Centre for Resistance Research (TZR), School of Veterinary Medicine, Freie Universität Berlin, Berlin, Germany; 3 Present Address: Institute of Microbiology, University of Veterinary Medicine Hannover, Bischofsholer Damm 15, 30173 Hannover, Germany

**Keywords:** Persisters, ROS, LepA, Prophages, *Salmonella*, Microbiology, Molecular biology

## Abstract

Bacterial persister cells are known to survive antibiotic treatment in the absence of typical resistance mechanisms. Persisters do not grow in the presence of antibiotics but can resume growth when antibiotic treatment is ceased, persisters are therefore often associated with chronic infections and treatment failure. In previous studies, it was shown that a *lepA* deletion strain of *Escherichia coli* was more tolerant to different classes of antibiotics due to reduced formation of reactive oxygen species (ROS). LepA is a highly conserved protein involved in translation, but the details of its role in the various mechanisms it participates in remain unclear. In this study, we performed killing assays to assess persister survival in a *lepA* deletion strain of *Salmonella* Typhimurium and FACS analysis to measure ROS formation in response to ciprofloxacin, β-lactam, and aminoglycoside antibiotics, representing three different classes of bactericidal drugs. Our results suggest that deletion of *lepA* reduces ROS formation and may increase persister survival after ciprofloxacin treatment, possibly due to reduced prophage induction. In contrast, reducing oxidative stress with the antioxidant glutathione was associated with increased persister formation and a larger subpopulation of wild type bacteria lacking prophage induction following ciprofloxacin treatment, whereas no additional beneficial effect was observed in the *lepA* mutant. These findings are consistent with a link between ROS-associated prophage induction and persister cell levels, providing a cautious perspective on how drug-induced ROS may influence bacterial survival in strains carrying prophages.

**Supplementary Information:**

The online version contains supplementary material available at 10.1038/s41598-026-47552-0.

## Introduction

A small subpopulation of otherwise antibiotic-susceptible bacteria can survive treatment with antibacterial agents without acquiring genetic resistance^1^. These cells, called persisters, are dormant or slow-growing bacteria that tolerate antibiotics but regain susceptibility once the antibiotic pressure is removed. A hallmark of antibiotic persistence is a biphasic killing curve, consisting of a rapidly dying majority of the bacterial population and a slowly dying small subpopulation. This phenomenon known as antibiotic persistence had been described in the 1940s by Joseph Bigger who found that exposure of *Staphylococcus aureus* to penicillin failed to eradicate the inoculated culture^2^. Classical genetic resistance mechanisms were not responsible for the survival because re-inoculation of the persistent bacteria led to the same degree of killing as for the original population. Long neglected, research on persister cells revived again in the 1980s, when Moyed et al. identified *hipA* as a major factor for increased tolerance to ampicillin and fosfomycin^3^. Since then, various mechanisms have been described as to how persisters survive lethal exposure to antibiotics, including the SOS or oxidative stress response^4,5^, elevated levels of (p)ppGpp, slow growth^6^, activation of toxins and nutrient limitation^7–10^, or reduced concentrations of ATP^11–13^, although the involvement of low ATP remains a matter of debate^10,11,14,15^. However, another important aspect for persister cell formation is the induction of endogenous prophages, which can also bias the survival of bacteria after antibiotic treatment^10,16^. The *Salmonella enterica* subsp. *enterica* serovar Typhimurium strain ATCC 14028 (*S*. Typhimurium) harbors four inducible prophages designated Gifsy-1, Gifsy-2, Gifsy-3, and ST64B^17–19^. Previously, we had demonstrated that Gifsy-1 is the strongest derepressed prophage upon ciprofloxacin treatment and that the expression of its lysis genes are mainly responsible for the increased initial killing and reduced persister cell formation upon DNA damage^16^. Upon salmonellosis, ciprofloxacin is often the first-choice antibiotic, particularly for elderly or immunocompromised individuals, in whom a *Salmonella* infection can be life-threatening^20^. As a DNA-damaging antibacterial agent, ciprofloxacin also has the potential to induce prophages and enhance bacterial killing in vitro.These observations are also consistent with other studies supporting the significance of resident prophages in terms of antibiotic susceptibility, for example, *Pasteurella haemolytica* and *Escherichia coli* are more sensitive to the DNA gyrase inhibitor danofloxacin when carrying inducible prophages^21^.

LepA is a highly conserved protein found in bacteria, mitochondria and chloroplasts; however, the exact function of LepA is still under discussion. Several functions for LepA have been proposed, such as translational proof-reading, allowing the ribosomes a more accurate elongation of the nascent polypeptide chain^22,23^. Other studies suggested that LepA contributes to the initiation of translation or is involved in the maturation of ribosomes^24–26^. LepA has been found to improve growth of *Helicobacter pylori* under acidic conditions, and increased the resistance of *E. coli* against ionic stress^27,28^. However, under standardized laboratory conditions, LepA is dispensable for bacterial growth in *E. coli*^22,29^. Further phenotypes associated with *lepA* deletion include increased production of the calcium-dependent antibiotic (CDA) in the Gram-positive bacterium *Streptomyces coelicolor* without affecting bacterial growth, suggesting a regulatory role of LepA during translation^30^. In addition, deletion of *lepA* in *Mycobacterium tuberculosis* has been linked to increased rifampicin tolerance, which was associated with transcriptional upregulation of *rpoB* and impaired porin synthesis, potentially reducing rifampicin uptake^31,32^. Furthermore, treatment failure in rifampicin-tolerant clinical mycobacterial isolates has been associated with mutations in *lepA*, indicating that *lepA* may play an important role from a clinical perspective^31^. These observations indicate that LepA influences translational processes not only in Gram-negative bacteria but also in Gram-positive species. Interestingly, deletion of *lepA* in *E. coli* conferred increased drug tolerance against β-lactam antibiotics, aminoglycosides, and fluoroquinolones, presumably by reducing the formation of reactive oxygen species (ROS)^33^. ROS has been found to contribute to bacterial killing by antibiotics due to the oxidative damage of proteins and DNA^15,34–38^. It has been demonstrated that different classes of bactericidal antibiotics, including fluoroquinolones, can stimulate the formation of reactive oxygen species by inducing metabolic perturbations involving the TCA cycle, NAD metabolism, nucleotide metabolism, and energy metabolism^15,37^. As a consequence, there is an increased activity of central carbon metabolism and enhanced formation of NADH, which in turn stimulates respiratory activity and oxygen consumption^15,34,39^. This increases the risk of superoxide formation and leads to damage of iron-sulfur clusters in proteins, releasing iron into the cytosol. There, the Fenton reaction generates highly reactive hydroxyl radicals, which cause DNA damage and replication errors, thereby contributing to bacterial cell death upon antibiotic treatment. Therefore, limiting the formation of ROS is beneficial for the survival of bacteria because it reduces cellular damage, whereas increasing the formation of ROS could improve the efficiency of antibiotics and reduce the emergence of drug-tolerant persisters^15^.

In this study, we investigated the role of LepA and the involvement of the endogenous prophages in antibiotic killing in *Salmonella enterica* Serovar Typhimurium. Our results revealed that reduced ROS formation in a *lepA* deletion mutant interfered with prophage induction compared to the wild type. However, only the fluoroquinolone ciprofloxacin, but not β-lactams or aminoglycosides, induced ROS and the SOS response to levels sufficient to trigger prophage induction, resulting in reduced persistence of the wild type compared to the *lepA* mutant. The results of this study therefore link antibiotic-induced ROS formation with prophage induction, and sheds new light upon ROS-dependent antibiotic killing.

## Results

### Persister rate of Δ*lepA* after treatment with three different classes of antibiotics

**Fig. 1:**
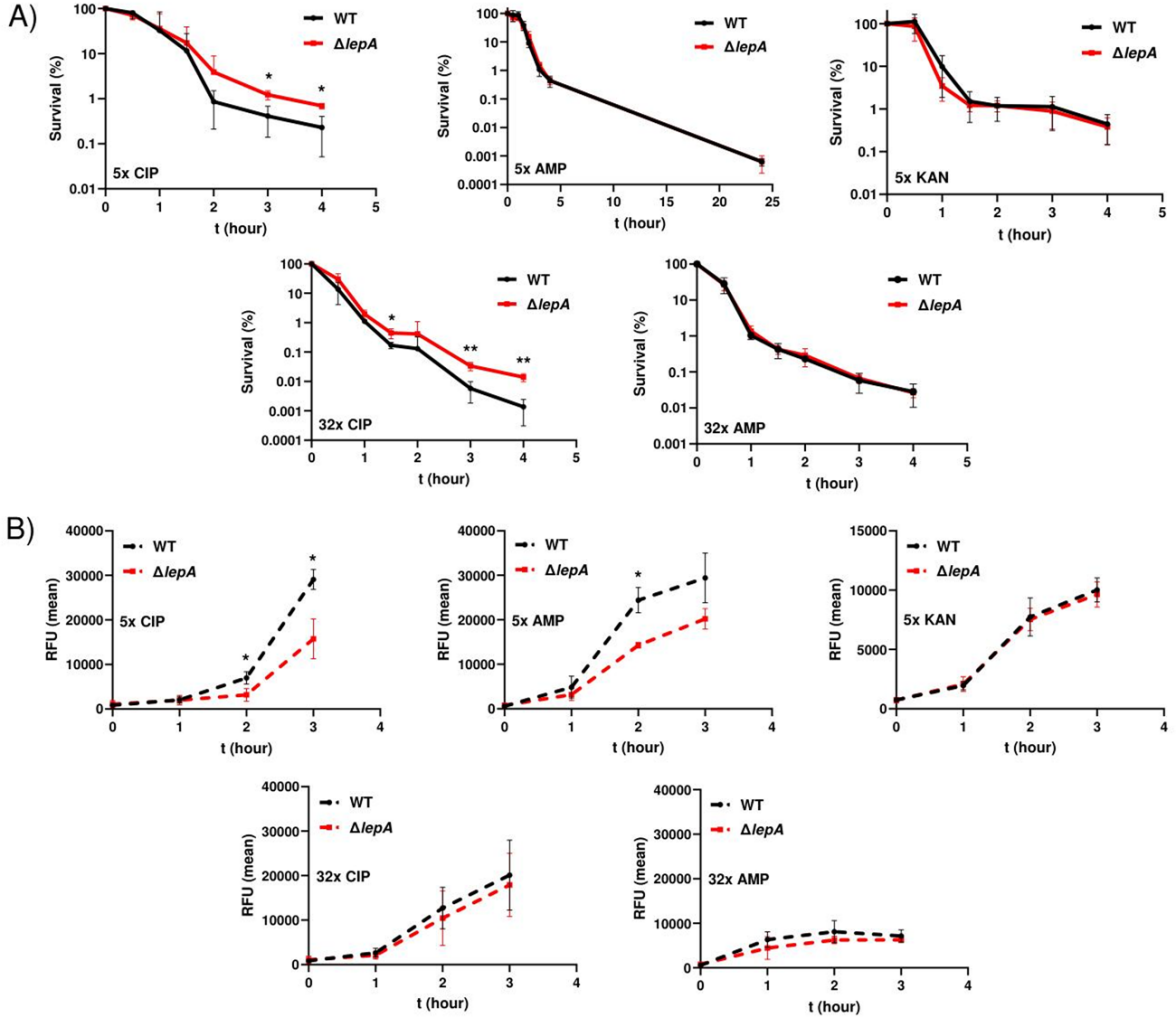
Persister assays with *Salmonella* Typhimurium and three classes of antibiotics and the corresponding formation of drug-induced hydroxyl radicals. (**A**) Exponentially growing bacteria (8640 = WT; SB6 = *lepA*) were treated with either ciprofloxacin (CIP), ampicillin (AMP) or kanamycin (KAN) with 5x MIC or 32x MIC respectively (ciprofloxacin: 5x MIC = 0.625 µg/ml; 32x MIC = 4 µg/ml, 5x MIC = 3.9 µg/ml; 32x MIC = 25 µg/ml and kanamycin 5x MIC = 15.6 µg/ml). The survival was calculated as percentage and was normalized to the start CFU. (**B**) To determine ROS formation the wild type and *lepA* mutant were exposed to the indicated antibiotics and subsequently dyed with HPF, which produces a green fluorescence signal in the presence of hydroxyl radicals. The average fluorescence signal (FITC) of the bacterial population was determined by FACS and plotted against the time. At least three independent experiments were performed. Data are presented as mean ± standard deviation (SD). Statistical significance was assessed using an unpaired two-tailed t-test with Welch’s correction. A *p* value < 0.05 was considered statistically significant (*p < 0.05; **p < 0.01).

Prior studies indicated that deletion of *lepA* in *E. coli* significantly increased drug tolerance against three different classes of antibiotics^33^. In order to determine the survival and persister cell formation of a *lepA* deletion (Δ*lepA*) mutant in *S*. Typhimurium, we conducted time-kill assays with ciprofloxacin, ampicillin and kanamycin at 5x MIC and 32x MIC, respectively, as previously described^33^. An aliquot was collected prior to the addition of antibiotics to determine baseline CFU values, which served as the 100% survival reference. Following antibiotic treatment, samples were collected at the indicated time points, and CFUs were determined to calculate percent survival relative to the pre-treatment baseline. As shown in Fig. 1A, exposure to ampicillin or kanamycin resulted in typical biphasic killing curves, a hallmark of persister cell formation. However, no difference between the wild type and the *lepA* mutant was observed, regardless of the MIC. Note that 32x MIC of kanamycin resulted in complete eradication of *S.* Typhimurium within the first two hours, therefore time-kill assays with 32x MIC kanamycin were not performed. In contrast to ampicillin and kanamycin, persister cell formation of the *lepA* deletion mutant was significantly increased compared to the wild type strain after treatment with ciprofloxacin (Fig. 1A). Interestingly, exposure to 5x ciprofloxacin led to a three-phase killing curve with a delay of 60 min before entering into the second phase with rapid killing of drug sensitive bacteria followed by the third phase (plateau), represented by slow killing of persister cells (Fig. 1A). This phenomenon was very similar to our previous study regarding prophage-dependent persister cell formation, with the bimodal curve of the wild type, prophage-harboring strain becoming a triphasic killing curve in prophage-free strains^16^. We also tested whether impaired resuscitation was responsible for the increased persister cell survival observed in the Δ*lepA* mutant. To address this, we performed a resuscitation assay using pyruvate to metabolically stimulate ciprofloxacin-treated bacteria^40^. After four hours of ciprofloxacin treatment, the bacteria were additionally incubated in M9 medium supplemented with 50 mM pyruvate prior to plating on LB agar plates. However, this additional treatment prior to plating decreased persister cell survival in both strains, fivefold in the wild type and twofold in the Δ*lepA* mutant, suggesting that viable but nonculturable cells (VBNC) play a negligible role in the observed phenotypes (Supp Fig. S1A).

Furthermore, deletion of *lepA* also improved survival and persister cell formation compared to the wild type at 32x MIC (Fig. 1A). In contrast to 5x MIC, treatment with 32x MIC ciprofloxacin resulted in a less pronounced triphasic killing curve, more closely resembling a typical biphasic curve suggesting that prophages may be less important for the differences between the wild type and the mutant at 32x MIC ciprofloxacin (Fig. 1A). Furthermore, in contrast to *E. coli*, where deletion of *lepA* resulted in increased drug tolerance to a variety of antibiotic classes, persister survival in *S*. Typhimurium was only increased upon fluoroquinolone treatment, indicating that *lepA* is not a general persister factor in *Salmonella*.

### Hydroxyl radical formation after exposure to antibiotics

The involvement of reactive oxygen species in bacterial killing during antibiotic therapy is a well-documented phenomenon^15,34–39^. The drug-induced perturbation of bacterial metabolism includes an increased activity of central carbon metabolism and NAD metabolism as well as depletion of the nucleotide pools, and augmented oxygen consumption and the formation of superoxide, which in turn damages the iron-sulfur clusters of proteins^15,34,37,39^. The released iron can react with hydrogen peroxide to form reactive hydroxyl radicals, which can cause DNA double strand breaks by oxidizing or hydroxylating the DNA^36,41,42^. To investigate a possible correlation between persister cell formation and hydroxyl radical formation for the wild type and the corresponding *lepA* mutant after antibiotic treatment, we performed FACS analysis using the hydroxyl radical specific dye hydroxyphenylfluorescein (HPF)^15^. Logarithmic growing bacteria were treated with the respective antibiotic as before for the time-kill assays, and the formation of hydroxyl radicals was determined to test whether there was a correlation between the formation of hydroxyl radicals and persister survival. All antibiotics induced the formation of hydroxyl radicals (Fig. 1B); however, a difference between the wild type and the *lepA* mutant was only detected after treatment with ciprofloxacin and ampicillin at 5x MIC, but not at 32x MIC or after exposure to kanamycin (Fig. 1B). Induction of hydroxyl radicals after one hour of treatment correlated with the onset of bacterial killing after ciprofloxacin exposure. However, the increase in hydroxyl radical production in the *lepA* mutant was lower compared to that in the wild type strain, consistent with the reduced killing observed at later time points. These results indicate that hydroxyl radicals may play a role in the killing mechanism, which appears to be more pronounced in the wild type than in the *lepA* mutant under 5x MIC ciprofloxacin treatment. Without antibiotics, only a weak ROS signal was detected, and no difference between the two strains was observed during exponential growth (Supp Fig. S1B).

**Fig. 2:**
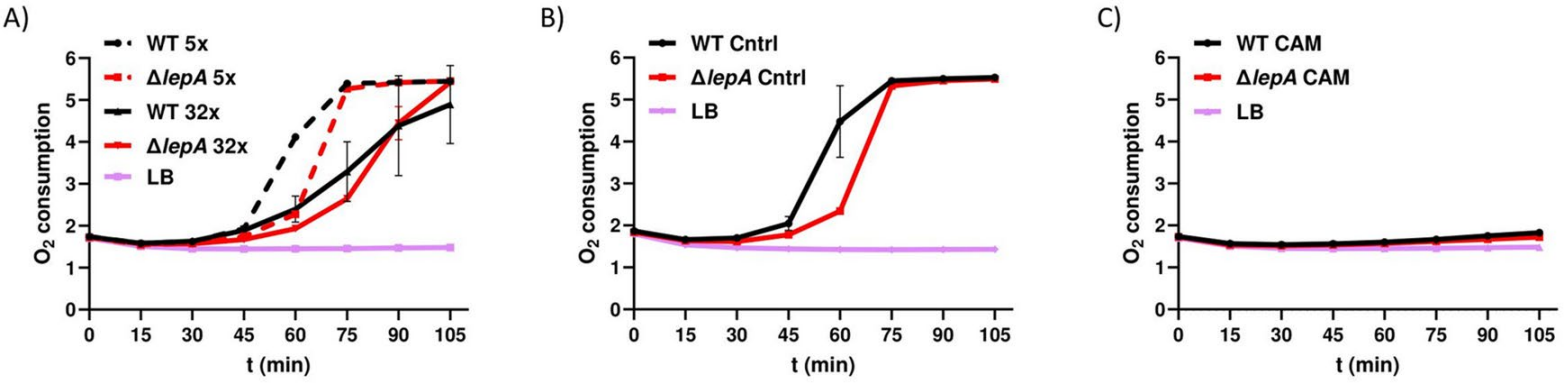
Oxygen consumption of *Salmonella* Typhimurium and the *lepA* mutant. Respiratory activity was assessed using Oxoplates at 5x MIC and 32x MIC concentrations of ciprofloxacin. LB medium was covered with paraffin oil to prevent gas exchange, allowing measurement of oxygen consumption by the bacteria. (**A**) CIP-treated bacteria at the indicated MIC; (**B**) control without antibiotics and (**C**) bacteria exposed to 5x MIC chloramphenicol (Cam) to confirm the absence of oxygen consumption as an additional control. Experiments were performed in triplicate, and one representative result is shown.

LepA is a translational factor involved in the synthesis of new proteins. Therefore, deletion of *lepA* could impair translation fidelity or efficiency. The bacterial electron transport chain, located in the inner membrane, consists of several intricate enzyme complexes, where even subtle defects in translation could accumulate and result in reduced respiratory activity and oxygen consumption, a condition that may lower oxidative stress during antibiotic treatment. To assess this, we measured oxygen consumption following treatment with ciprofloxacin at 5x MIC and 32x MIC. When treated with 5x MIC ciprofloxacin, O₂ consumption increased more rapidly in the wild type strain than in the Δ*lepA* mutant between 45 and 60 min, as indicated by the steeper slope of the oxygen consumption curve over time (Fig. 2A; slope angle ~ 50% for WT vs. ~17% for the Δ*lepA* mutant). Later, the oxygen consumption of the *lepA* mutant became comparable to that of the wild type, indicating that only the onset of respiratory activity is disturbed in the *lepA* mutant. A similar effect was observed in the absence of antibiotics, suggesting that the initial deficiency in respiratory activity is a general feature of the *lepA* mutant and not triggered by ciprofloxacin (Fig. 2B). We also treated the bacteria with 32x MIC ciprofloxacin and observed only minor differences between the two strains (Fig. 2A). As a control, we used 5x MIC chloramphenicol to inhibit respiration (Fig. 2C). Furthermore, the oxygen consumption of both strains was generally lower at higher ciprofloxacin concentrations, consistent with reduced ROS formation under conditions of high antibiotic stress^43,44^. Consequently, we concentrated on treatment with 5x MIC ciprofloxacin to assess how elevated ROS levels in the wild type influence persister cell formation.

### Weak induction of prophages in the *lepA* mutant increases persister cell survival

**Fig. 3:**
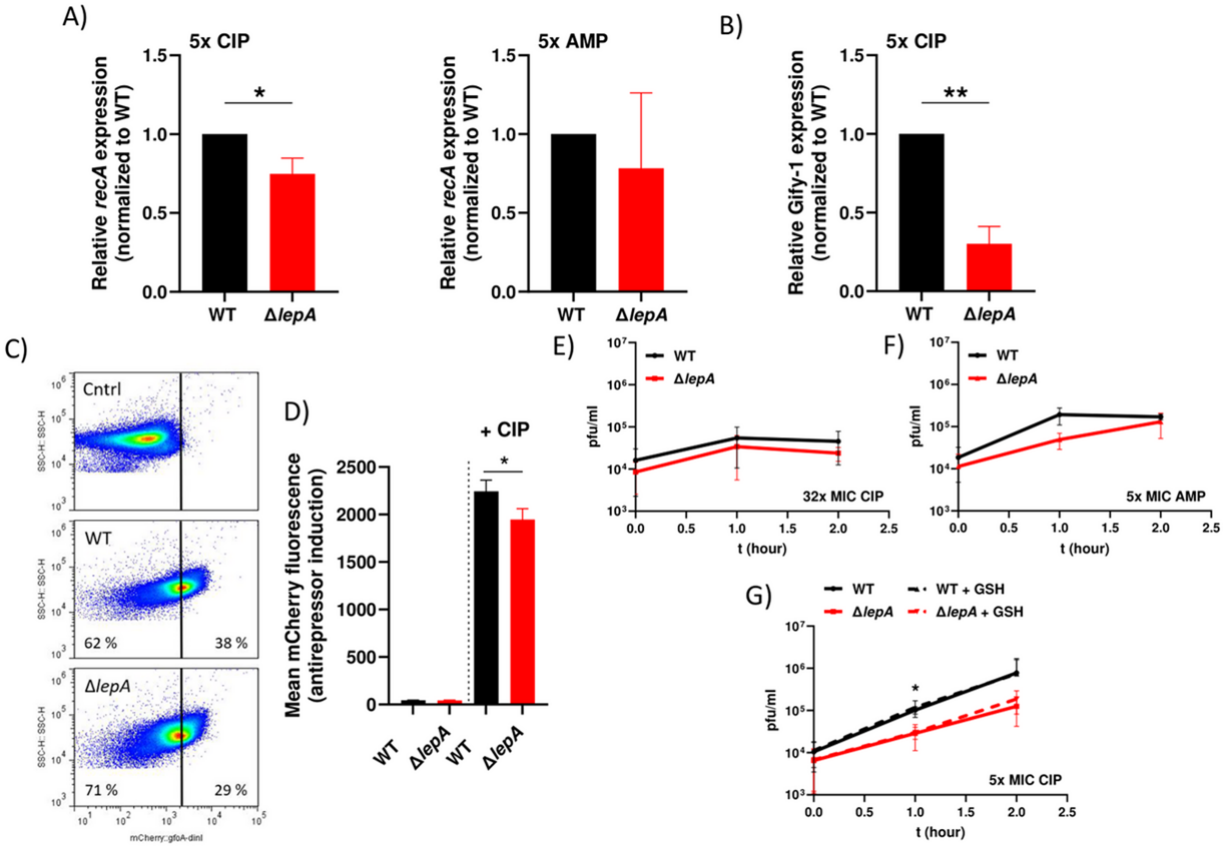
Detection of *recA* and phage induction after drug treatment. Exponential growing bacteria (WT = 8640; Δ*lepA* = SB6) were treated with ciprofloxacin or ampicillin (5 x MIC) for 2 h before RNA extraction for quantitative PCR. Illustrated is the induction of (**A**) *recA*, normalized to the housekeeping gene *trpA* for each strain, followed by calculation of the expression ratio between the wild type and the *lepA* mutant (mutant vs. wild type). (**B**) The same approach was used to detect transcriptional activation of STM2605, encoded within Gifsy-1. (**C**) The antirepressor (*gfoA*) and the damage inducible gene (*dinI*) were replaced by the fluorescent protein mCherry to measure the SOS response-dependent induction of Gifsy-1 upon treatment with 5x MIC of ciprofloxacin for three hours (mCherry::gfoA-dinI = SB287; Δ*lepA* mCherry::gfoA-dinI = SB283). The solid line represents the gating strategy. Cntrl (control) stands for the strain 11326 (ΔGifsy-2 ΔGifsy-3 ΔST64B) without mCherry. One representative example of a dot plot is shown. (**D**) The figure represents the mean fluorescence signal of the antirepressor mCherry reporter from three independent experiments, with and without ciprofloxacin (5x MIC). **(E)** Plaque assays with MS1487 and SB532 were performed using 32x MIC ciprofloxacin or (**F**) 5x MIC ampicillin. (**G**) Plaque assay with 5x MIC ciprofloxacin and 2 mM glutathione. At least three independent experiments were performed. Data are presented as mean ± standard deviation (SD). Statistical significance was assessed using an unpaired two-tailed t-test with Welch’s correction. A *p* value < 0.05 was considered statistically significant (*p < 0.05; **p < 0.01).

In a previous study, we performed killing assays of prophage-free and ΔGifsy-1 *S.* Typhimurium strains challenged with ciprofloxacin^10,16^. Strain 14028 carries at least four SOS-controlled prophages, with Gifsy-1 being the most strongly derepressed^10,16,45,46^. Deletion of all four prophages, or of Gifsy-1 alone, delayed the initial killing and increased persister survival^16^. Based on these prior observations, we hypothesized that deletion of *lepA* could reduce the induction of endogenous prophages, potentially contributing to the slower bacterial killing observed after ciprofloxacin treatment. Gifsy-1 induction is controlled by RecA, the activator of the DNA repair system^47^. Therefore, we examined the transcriptional upregulation of *recA* as an indicator of the SOS response following ciprofloxacin treatment. After two hours of treatment with 5x MIC ciprofloxacin, RNA was extracted and used for RT-qPCR to determine the level of *recA* expression. We observed that *recA* transcription was approximately 25% less strongly induced in the *lepA* mutant compared to the wild type (Fig. 3A). As a control, we exposed both strains to ampicillin, which targets the cell wall^48^. As before, the bacteria were treated with ampicillin for two hours to measure the induction of *recA* expression. However, unlike ciprofloxacin treatment, no difference was observed between the two strains regarding induction of the SOS response (Fig. 3A). The differential effects of antibiotics on persister cell formation and bacterial killing are supported by experiments using a non-cleavable LexA/SOS-repressor lexA3(ind^-^) strain^44,45^. In this strain, ciprofloxacin treatment reduced persister cell formation, consistent with SOS-dependent regulation of persistence, whereas the bactericidal effects of ampicillin and kanamycin were unaffected (Supp Fig. S2). This strengthens the assumption that DNA damage induced under these conditions is not sufficient to reduce persister cell survival via prophage activation when cells are treated with aminoglycosides or β-lactam antibiotics, in contrast to fluoroquinolones. In addition, to measure induction of Gifsy-1, the strongest derepressed prophage in our strain upon treatment with ciprofloxacin, we performed qPCR to determine the transcriptional upregulation of STM2605, a gene encoded within Gifsy-1^49^. Transcriptional induction of Gifsy-1 was approximately 70% higher in the wild type compared to the *lepA* mutant following treatment with 5x MIC ciprofloxacin (Fig. 3B). Motivated by these results, and to further support our hypothesis that prophage induction was responsible for the difference between the wild type and the *lepA* mutant after treatment with ciprofloxacin, we constructed a mutant in which the SOS-dependent (LexA-repressed) gene *gfoA* (Gifsy-1 anti-repressor and essential for prophage induction^45^ and the adjacent gene *dinI* of Gifsy-1 were replaced by sequences encoding the fluorescent protein mCherry^45^. Here, activation of the SOS-response (LexA inactivation) should result in increased expression from the *gfoA* promoter and hence mCherry expression, serving as a reporter for the SOS response and increased expression of the GfoA anti-repressor, a prerequisite for initiation of Gifsy-1 induction^10^. The constructed strains were subjected to treatment with 5x MIC ciprofloxacin for three hours. As shown in Fig. 3C and D, treatment of both strains resulted in an elevated mCherry signal, with the wild type strain showing a higher signal compared to the *lepA* mutant, indicating a stronger prophage activation in the wild type, and suggesting that derepression, i.e. increased SOS-response-dependent expression of the GfoA anti-repressor, was higher in the wild type in response to ciprofloxacin, and by implication that the *lepA* mutant would have a reduced prophage activation during the SOS response. Furthermore, the subpopulation without induction of prophages was higher in the *lepA* mutant, which was also consistent with a larger subpopulation of the *lepA* mutant with reduced ROS formation. As a control, we also treated the reporter strains with 5x MIC ampicillin, but observed no changes in *gfoA* expression (Supp Fig. S3).

Next, we performed plaque assays to assess the formation of functional Gifsy-1 prophages following treatment with 5x MIC or 32x MIC ciprofloxacin and 5x MIC ampicillin. Exposure to 32x MIC ciprofloxacin resulted in maximal plaque formation after one hour, which then remained stable, indicating that no further phage production occurred beyond this point (Fig. 3E). Importantly, no differences in plaque formation were observed between the two strains at 32x MIC ciprofloxacin. A similar pattern was observed with 5x MIC ampicillin, where plaque formation initially increased and subsequently plateaued (Fig. 3F). Notably, plaque formation increased more slowly in the Δ*lepA* mutant compared to the wild type, which may be due to reduced ROS formation during drug treatment. However, after two hours of ampicillin treatment, plaque numbers were indistinguishable between the two strains. In contrast, exposure to 5x MIC ciprofloxacin led to significantly higher plaque formation in the wild type after one hour compared to the Δ*lepA* mutant (Fig. 3G). Plaque numbers continued to increase over time and remained consistently higher in the wild type, consistent with FACS analysis using the mCherry antirepressor reporter strains. These results indicate that under low ciprofloxacin exposure, prophages are continuously induced, whereas treatment with ampicillin or high-dose ciprofloxacin results in cessation of prophage production after one hour.

### Hydroxyl radical dependent induction of prophages

**Fig. 4:**
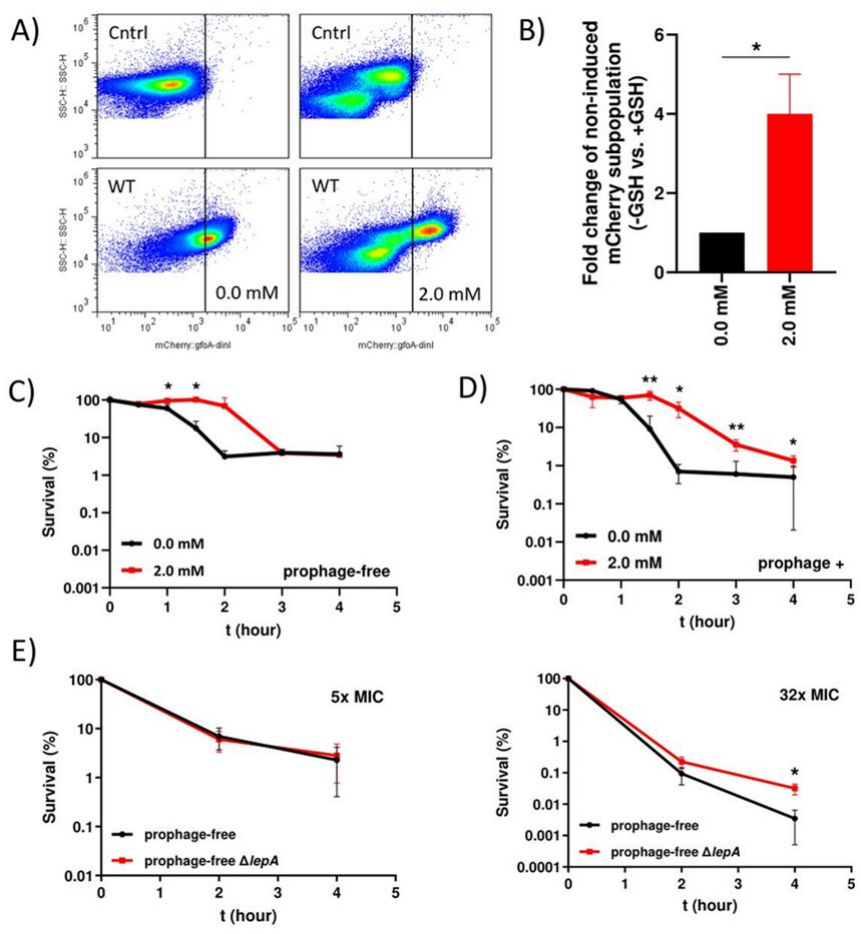
Ciprofloxacin treatment with or without the antioxidant glutathione. (**A**) Detection of prophage (Gifsy-1) anti-repressor induction in strain SB287 (8640 *mCherry::gfoA-dinI*) after exposure to 5x MIC ciprofloxacin for three hours, either with the indicated concentration of glutathione or without. The solid line represents the gating strategy. “Cntrl” (control) refers to strain 11326 lacking mCherry. One representative dot plot is shown. (**B**) The subpopulation without induction of the prophage anti-repressor shown in panel A was quantified after the addition of glutathione and normalized to the culture without glutathione (0.0 mM). (**C**) The prophage-free variant (11126) was treated with 5x MIC ciprofloxacin with or without the indicated concentration of glutathione. (**D**) The wild type strain (prophage-positive, 8640) was treated under the same conditions. **(E)** The prophage-free *lepA* mutant (SB421) was treated with 5x and 32x MIC ciprofloxacin, and survival was compared to the prophage-free wild type strain (11126). At least three independent experiments were performed. Data are presented as mean ± standard deviation (SD). Statistical significance was assessed using an unpaired two-tailed t-test with Welch’s correction. A *p* value < 0.05 was considered statistically significant (*p < 0.05; **p < 0.01).

Based on the observations that deletion of *lepA* resulted in reduced formation of hydroxyl radicals/ initial respiratory activity, SOS response, *gfoA* expression, decreased killing after ciprofloxacin treatment and plaque formation, we hypothesized that these outcomes were linked. The combination of hydroxyl radicals and ciprofloxacin appeared to increase the induction of the SOS response and thus enhanced the activation of the prophages in the wild type, ultimately leading to a more efficient killing and reduced persister cell survival. To further examine the role of ROS and the SOS response in prophage activation, we treated the wild type and the *lepA* mutant with 5x MIC ciprofloxacin in combination with the antioxidant glutathione and assessed plaque formation. Contrary to our expectations, glutathione had no effect on plaque formation within the first two hours (Fig. 3G), in either the wild type or the mutant. To investigate potential effects on subpopulations, we utilized our antirepressor reporter strain. Bacteria were treated with 5x MIC ciprofloxacin and glutathione, followed by FACS analysis. Co-treatment with glutathione increased the subpopulation that showed no induction of the antirepressor (Fig. 4A). This subpopulation was four times larger compared to the culture without glutathione (Fig. 4B). Interestingly, glutathione supplementation also increased the intensity of the mCherry signal, as shown by a rightward shift of a large bacterial population in the dot plot (Fig. 4A). These findings indicate that glutathione treatment affects subpopulations differently, with some cells showing reduced mCherry signal (i.e., weak prophage induction) and others showing increased induction. Because plaque assays measure the overall population response, they may not reliably detect ROS-mediated prophage induction at the level of persister cells. To directly assess the impact of glutathione on persister cells without interference from prophage induction, we therefore performed experiments using our prophage-free strain^10,16^. Treatment of the prophage-free strain with ciprofloxacin in combination with glutathione slowed the initial killing but had no effect on persister cell levels (Fig. 4C). In contrast, in the prophage-positive wild type treated with 5x MIC ciprofloxacin and glutathione, an increased fraction of persister cells was observed (Fig. 4D). Furthermore, no beneficial effect of ROS quenching by glutathione was observed for the *lepA* mutant. In contrast, the persister fraction of the *lepA* mutant decreased upon glutathione addition (Supp Fig. S4). To confirm that prophages account for the observed difference in persister levels between the wild type and the *lepA* mutant, we deleted *lepA* in the prophage-free background and performed killing assays with 5x and 32x MIC ciprofloxacin. As expected, at 5x MIC the difference between the wild type and mutant disappeared in the absence of prophages (Fig. 4E). However, at 32x MIC, the *lepA* mutant still showed a higher fraction of persister cells, suggesting the existence of a ROS-and prophage-independent mechanism (Fig. 4E). These results suggest that prophages may be induced by ROS formation at 5x MIC ciprofloxacin and could contribute to the lower persister cell fraction observed in the wild type compared to the *lepA* mutant.

**Fig. 5:**
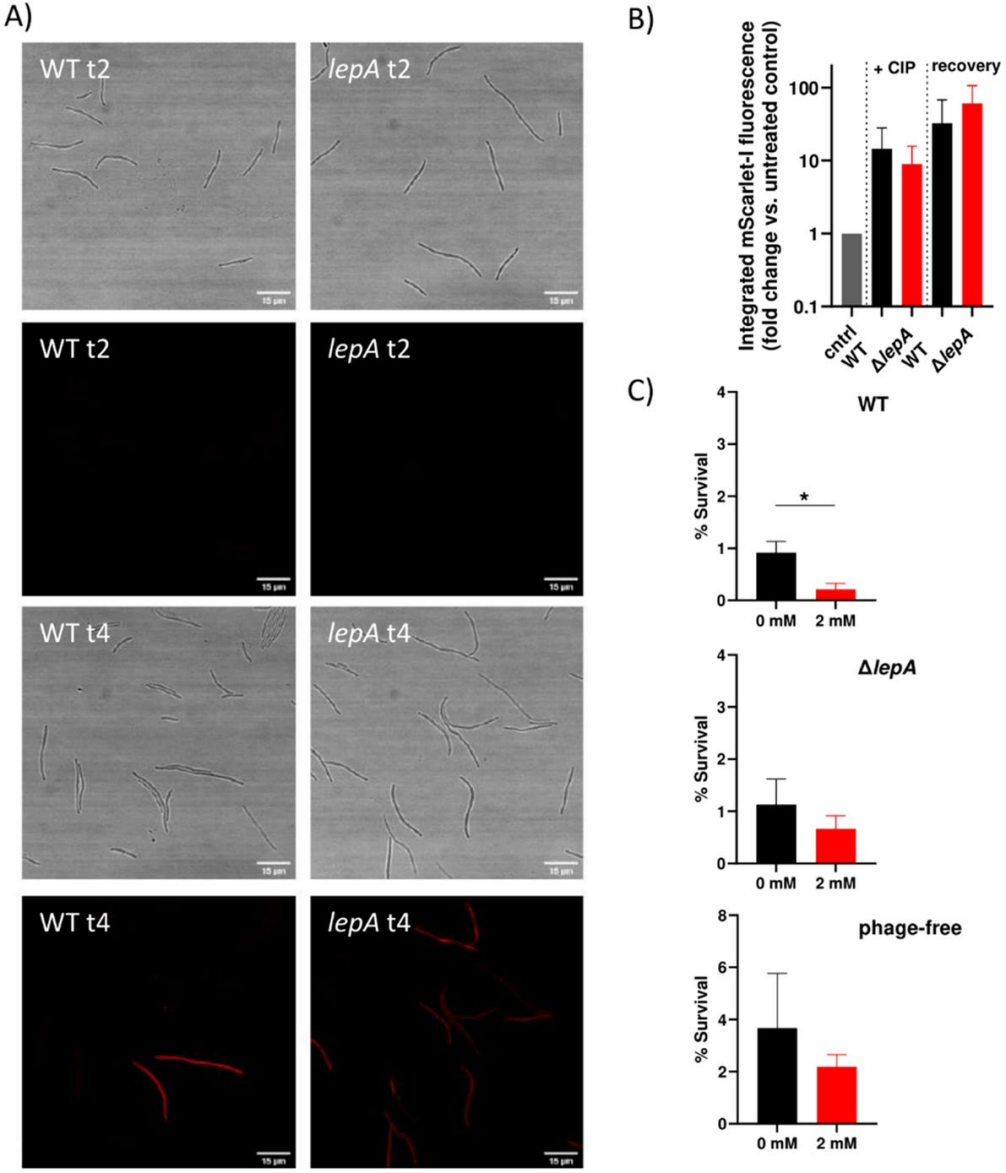
ROS stress during the recovery phase. (**A**) Microscopic images of the wild type and *lepA* mutant. To determine catalase activity, *katG* was fused to mScarlet-I (WT *katG*-mScarlet-I = SB531; *lepA katG*-mScarlet-I = SB501). Bacteria were treated for two hours with 0.625 µg/ml ciprofloxacin in liquid LB (5x MIC), subsequently washed, and spotted onto LB agarose pads. After spotting on LB agarose pads and after two hours of recovery, the mScarlet-I signal was visualized. t2 marks the beginning of the recovery phase (after 2 h of treatment), whereas t4 refers to two hours of recovery following the removal of ciprofloxacin. Bright-field and fluorescence images are shown. (**B**) Quantification of the mScarlet-I signal after imaging. “+CIP” refers to ciprofloxacin treatment, and “recovery” indicates two hours of recovery on LB agarose pads without antibiotics. “cntrl” represents the untreated control cultures (supplementary Fig. S5), to which the mScarlet signal of ciprofloxacin-treated cells was normalized for the respective strain to calculate the fold change. (**C**) Bacteria (wild type = 8640; *lepA* = SB6; prophage-free = 11126) were treated for four hours with ciprofloxacin and subsequently plated on LB agar supplemented with 2 mM glutathione. At least three independent experiments were performed. Data are presented as mean ± standard deviation (SD). Statistical significance was assessed using an unpaired two-tailed t-test with Welch’s correction. A *p* value < 0.05 was considered statistically significant (*p < 0.05).

In addition, we also investigated the impact of ROS formation during the recovery phase. We fused *katG*, which encodes the hydrogen peroxide-detoxifying enzyme catalase, to the fluorescent protein mScarlet-I. Bacteria were treated with ciprofloxacin for two hours, followed by a two-hour recovery phase. After two hours of treatment in liquid medium, we observed a ten-fold increase in the mScarlet-I signal compared to the respective untreated control in both strains. After an additional two hours of recovery (following spotting of the bacteria onto LB agarose pads), catalase expression further increased in a subset of cells in both the wild type and the Δ*lepA* mutant, indicating that the bacteria experience also oxidative stress during the recovery phase (Fig. 5A and B). These findings are consistent with previous studies^50^. KatG activity was non-significantly higher in the *lepA* mutant during the recovery phase, suggesting that the mutant tends to detoxify hydrogen peroxide more efficiently (Fig. 5B). Furthermore, both strains show a typical response to DNA-damaging agents by forming long filamentous cells, indicating an active SOS response, which in turn interferes with cell division^51^. In contrast to untreated cells, which are short rod-shaped cells without any detectable mScarlet-I signal (Supp Fig. S5). To reduce ROS-mediated stress during recovery, we supplemented LB agar plates with 2 mM glutathione. Bacteria were treated with 5x MIC ciprofloxacin for four hours and subsequently plated on LB + glutathione agar. The addition of glutathione had no beneficial effect on persister cell survival in either the *lepA* mutant or the prophage-positive and prophage-free wild type strains (Fig. 5C). In contrast, persister cell survival of the prophage-positive wild type was significantly reduced (Fig. 5C). This suggests that ROS-mediated prophage induction likely occurs during antibiotic treatment rather than during the recovery phase, i.e., when cells begin to regenerate after stress. Furthermore, these results suggest that induction of the oxidative stress response may be beneficial during recovery from drug treatment, and that the addition of glutathione interferes with the cell survival process.

We were also interested in whether ROS formation can induce prophages in the wild type at 32x MIC ciprofloxacin and thereby reduce persister survival. Therefore, we treated the prophage-positive wild type and the corresponding prophage-free variant with 32x MIC ciprofloxacin in combination with glutathione. After three hours of treatment, persister cell formation increased 22-fold and 58-fold in the prophage-positive wild type when co-treated with 2 mM and 2.5 mM glutathione, respectively, in contrast to the prophage-free variant, which showed only a 10-fold increase in the persister fraction (Supp Fig. S6A). After four hours of treatment, survival increased 136-fold and 363-fold at 2 mM and 2.5 mM glutathione, respectively, when prophages were still integrated into the chromosome, compared to only a 30-fold increase when prophages were deleted (Supp Fig. S6B). These results indicate that ROS can also induce prophages at high ciprofloxacin concentrations and is responsible for the killing of a large subpopulation of the wild type. Thus, reducing oxidative stress with glutathione protects against prophage induction and can increase bacterial survival.

### Lambda mediated killing in *E. coli*

**Fig. 6:**
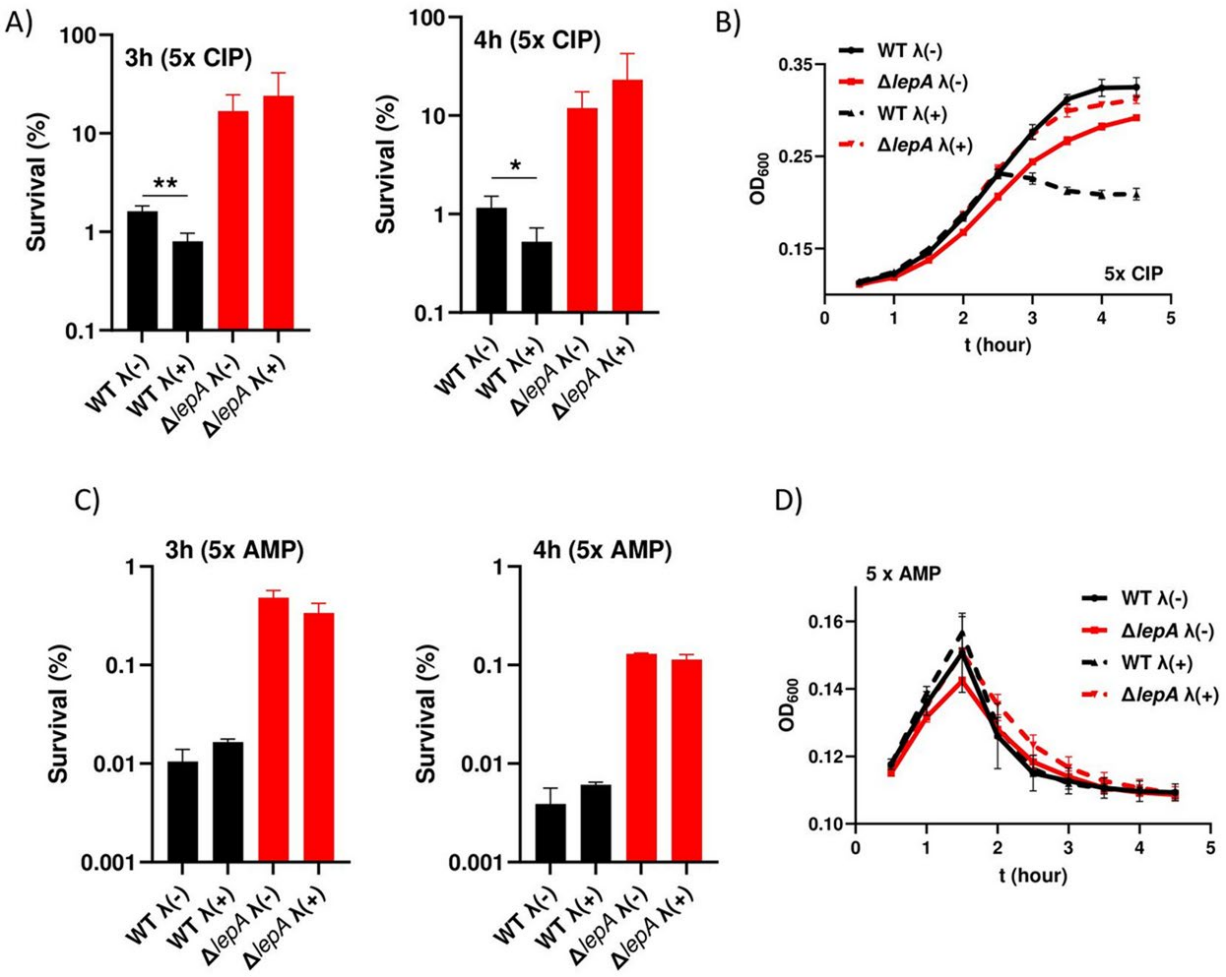
Killing assays in *Escherichia coli*. (**A**) Bacteria (145 = wild type; SB561 = *lepA* mutant; SB565 = wild type + lambda; SB564 = *lepA* mutant + lambda) were grown to exponential phase and then treated with 5x MIC ciprofloxacin (0.0195 µg/ml) for 3 and 4 h. (**B**) Lysis assays performed under the same conditions as in A). (**C**) Killing assays using 5x MIC ampicillin (15.625 µg/ml) for 3 and 4 h. **(D)** Corresponding lysis assays. At least three independent experiments were performed. Data are presented as mean ± standard deviation (SD). Statistical significance was assessed using an unpaired two-tailed t-test with Welch’s correction. A p value < 0.05 was considered statistically significant (*p < 0.05; **p < 0.01).

After establishing prophage-mediated killing in *S*. Typhimurium, we were also interested in whether prophages play a role in *E. coli* and its corresponding *lepA* mutant when treated with ciprofloxacin or ampicillin. Therefore, we used prophage-free variants of *E. coli* MG1655 as well as lambda-positive strains for killing assays. At 5x MIC ciprofloxacin, no difference was observed between the two *lepA* mutant strains, regardless of the presence of lambda (Fig. 6A). In contrast, in both wild type strains, persister cell formation was reduced upon ciprofloxacin treatment of the lambda-carrying variant, suggesting that ROS may contribute to prophage activation during drug treatment (Fig. 6A). In addition, exposure to ciprofloxacin resulted in clear lysis of the lambda-carrying wild type, whereas the corresponding *lepA* mutant showed no lysis (Fig. 6B). However, deletion of *lepA* still significantly increased drug tolerance, indicating the existence of a second, prophage-independent mechanism compared to the respective wild type. Upon treatment with ampicillin, lambda had no effect on persister cell survival, and deletion of *lepA* significantly increased survival compared to the respective wild type strains, indicating that lambda plays no role in survival following ampicillin exposure (Fig. 6C). This can be explained by the early lysis observed upon ampicillin treatment, which occurs approximately 90 min after drug exposure, leaving insufficient time for lambda prophage induction, phage assembly, and subsequent host lysis (Fig. 6D). In contrast, ciprofloxacin-treated wild types undergo lysis approximately two and a half hours after drug exposure, thereby allowing lambda prophage induction, phage assembly, and host cell lysis.

## Discussion

Drug resistance is recognized as one of the major health hazards of the coming decades. In addition to the acquisition of genetically defined resistance mechanisms, the inherent persistence of sub-populations of antibiotic-sensitive bacteria which survive antibiotic treatments not only contribute to therapeutic failure, but can eventually lead to increased tolerance, paving the way for resistance^52,53^. One approach to reduction of persister cell formation has been to exploit the formation of ROS during drug treatment. In several studies with various classes of bactericidal antibiotics, stimulating metabolic respiration, either by addition of carbon sources or electron acceptors, can lead to increased metabolic activity, ROS formation and drug efficiency^14,15,38,54^. In particular, the formation of reactive hydroxyl radicals lead to DNA breaks due to oxidation of the guanosine triphosphate pool (GTP) to 8-oxo-guanine^36^. Hydroxyl radicals are formed when hydrogen peroxide (H_2_O_2_) reacts with ferrous iron via the Fenton reaction^42^. This reaction is enhanced when NADH accumulates, as flavine reductase reduces FAD^+^ to FADH_2_, which is a potent electron donor for ferric iron turning it into ferrous iron^15,55^. In the present study, we observed variable ROS formation in *S*. Typhimurium following treatment with ciprofloxacin, ampicillin, and kanamycin. At 5x MIC, both ciprofloxacin and ampicillin strongly induced hydroxyl radicals (Fig. 1B). This may be explained by the antibiotic paradox, in which low to moderate drug concentrations allow RNA/DNA synthesis and translation, i.e., metabolic activity, thereby stimulating ROS formation, whereas high drug concentrations have the opposite effect^43,56^. LepA is a non-essential translation factor under optimal growth conditions; however, under stress conditions such as low temperature or ionic stress, LepA appears to enhance translation^28^. This may help explain why 32x MIC ciprofloxacin treatment resulted in increased persister survival in the *lepA* deletion mutant. Stress triggered by ciprofloxacin could be more efficiently compensated in the wild type with an active LepA protein, allowing translation to proceed more effectively than in the mutant. Antibiotic persistence is often associated with reduced metabolic activity, suggesting that LepA-mediated enhancement of metabolism in the wild type might lead to the killing of a subpopulation of potential persisters.

The effect of ROS formation upon treatment with antibactericidal drugs (5x MIC ciprofloxacin) was reduced when *lepA* was deleted in *S*. Typhimurium, which correlated with increased persister cell survival. It has been proposed that the lack of LepA increases the amounts of misfolded proteins^33^. This could disrupt the respiratory activity of bacteria. Therefore, we tested the respiratory activity of both strains. Regardless of the presence or absence of antibiotics, the deletion of *lepA* led to reduced initial respiratory efficiency (Fig. 2). It is possible that translation defects in the *lepA*-deficient strain impair the proper biosynthesis of electron transport chain components, therefore resulting in decreased oxygen consumption in a subset of bacteria. The reduced respiratory activity of the ∆*lepA* strain may consequently lead to lower hydroxyl radical production and an increased number of persister cells during antibiotic treatment. The importance of LepA on respiration is further suggested by studies in which deletion of the mitochondrial *lepA* homologue in mice (GUF1 or mtLepA), had been found to result in defects in oxidative phosphorylation^57^. Likewise, in yeast, deletion of GUF1 impairs the proper assembly of the cytochrome oxidase, which is also part of the respiratory electron transport chain^58^. Although absolute survival levels are low for the wild type and the *lepA* mutant, differences at this scale are biologically relevant in the context of persister cell biology. Even small surviving subpopulations can serve as reservoirs for regrowth, chronic infection, and the emergence of antibiotic resistance^59,60^. Importantly, intermittent or even a single treatment with fluoroquinolones or β-lactam antibiotics can increase the resistance or tolerance of surviving persisters to diverse classes of antibiotics, thereby making subsequent treatments more challenging^53,59^. An interesting example is the treatment of *Mycobacterium smegmatis* with rifampicin, which triggers elevated levels of ROS in rifampicin-tolerant cells that subsequently become resistant^61^. Considering that laboratory mycobacterial strains carry cryptic prophages that are unable to cause lysis, it is interesting to explore whether prophages could serve as potential adjuvants to combat mycobacterial infections by inducing host cell lysis upon activation^62^. Such an approach, in which bacteriophages and prophages are used to enhance antibiotic-mediated killing, is referred to as phage antibiotic synergy (PAS)^63,64^. In this strategy, either lytic bacteriophages are administered together with antibiotics to increase bacterial killing, or chromosomally integrated prophages are exploited to enhance the efficiency of DNA-damaging agents through lysis-mediated killing of the host. In this context, stimulation of ROS may further increase the antibacterial effect of antibiotics.

Here, the deletion of *lepA* only improved persister cell survival when bacteria were exposed to ciprofloxacin but not when treated with ampicillin or kanamycin, despite reduced ROS formation at 5x MIC ampicillin (Fig. 1). These results are partially consistent with a previous study on the drug tolerance of an *E. coli lepA* mutant^33^. In that prior study, LepA was proposed to be part of a ROS-mediated suicide pathway involving the MazF toxin that kills bacteria under stress conditions, such as when bacteria are treated with antibiotics, including ampicillin. However, deletion of all four prophages in *S*. Typhimurium resulted in the same killing kinetics for the wild type and the *lepA* mutant after treatment with 5x MIC of ciprofloxacin. This suggests that LepA is not part of a bacterial self-destructive pathway in *Salmonella* at low MIC. Rather, the increased persistence in the presence of the antibiotic appears to be a side effect of reduced ROS formation and the inefficient ROS-dependent prophage induction in the ∆*lepA* background. Second, we suggest that differences in the prophage repertoires of *S*. Typhimurium and *E. coli* may bias persister survival to different extents. In *E. coli*, prophages such as lambda or phi80 are induced due to activated RecA (RecA*) initiation of proteolytic inactivation of the prophage repressors^65,66^. In contrast, the prophages of *S*. Typhimurium are comparably weakly induced because activated RecA* must first promote self-cleavage of the SOS response repressor LexA, to derepress expression of the antirepressors of Gifsy, which in turn deactivate the actual prophage repressors^45,47^. These induction mechanisms would therefore also result in distinct killing kinetics depending on the class of antibiotic and the corresponding concentration. This assumption is consistent with the proposal of Harms et al.^67^, who examined toxin-mediated persister cell formation in cultures of *E. coli* infected with bacteriophage phi80^68^. The authors found that induction of phi80 was triggered upon ampicillin treatment in LB but not in minimal medium (supplemented with glucose), correlating with a stronger formation of ROS in LB compared to minimal medium^69^. In our study, lambda-positive strains showed reduced persister cell survival after exposure to ciprofloxacin, which may reflect the impact of prophage activation and lysis-mediated killing of the host. No detrimental effect was observed following ampicillin treatment, possibly due to early lysis caused by the antibiotic itself. This suggests that, even within the same bacterial species, prophage composition can have a significant impact on persister survival. Furthermore, no differences were observed between the two *lepA* mutants (lambda-positive and lambda-negative strains) when treated with 5x MIC ciprofloxacin, indicating that the level of DNA damage was too weak to induce prophage activation, which was confirmed by the absence of lysis in the corresponding assays (Fig. 6B). Considering that deletion of *lepA* reduces ROS formation, we conclude that the differences observed between the two wild type strains (lambda-positive and lambda-negative) result from enhanced prophage induction driven by increased ROS levels. This is further supported by studies showing that increasing concentrations of hydrogen peroxide and other reactive oxygen species correlate with enhanced lambda phage induction^70,71^. In addition, we examined ROS formation during the recovery phase and assessed the effect on persister survival by reducing oxidative stress with glutathione. For this purpose, bacteria were plated on LB agar supplemented with glutathione following ciprofloxacin treatment. However, survival was not increased in either the wild types (prophage-free and prophage-positive) or the *lepA* mutant. One possible explanation is that glutathione reduces the activity of OxyR, a transcriptional regulator that activates genes involved in hydrogen peroxide detoxification and, importantly in this context, can inhibit DNA replication via induction of Dps^72^. Dps, in turn, prevents cell division in bacteria carrying damaged DNA and may therefore reduce antibiotic-mediated killing during the recovery phase.

As mention above, ROS formation during drug treatment might be exploited to increase the efficiency of antimicrobial agents, in particular when inducible prophages are involved. Prophages also have the potential to accelerate bacterial killing, as observed for the prophage-free variant of our wild type strain. The initial killing phase was noticeably slower compared to the prophage-positive wild type (compare the killing slopes of the prophage-free and prophage-positive strains in Fig. 4C and D). This indicates that prophages may also play an important role during the early killing phase, when drug-susceptible bacteria are eliminated more efficiently. Rapid killing mediated by prophage activation might therefore contribute to reduced persister survival. This interpretation is consistent with our previous observation that deletion of all four prophages converted a typical biphasic killing curve into a triphasic one, due to deceleration of the initial killing phase^16^. However, it should also be considered that prophages serve as carriers for virulence and drug resistance genes by transduction^69^. Stimulation of ROS could trigger endogenous prophages carrying virulence and/or drug resistance genes or inadvertently package bacterial DNA into the phage capsid and transfer it to non-pathogenic strains. Transmission of resistance genes conferring ampicillin resistance has been demonstrated under laboratory conditions among *E. coli*^73^. Likewise, P22-like prophages ES18 and PDT17 transduced drug resistance genes encoding resistance against ampicillin, chloramphenicol and tetracycline from a multi drug resistant epidemic strain of *S*. Typhimurium DT104 to a susceptible recipient^74^. Furthermore, prophages are common among bacteria, and pathogens have a striking number of prophages integrated into their chromosomes^75,76^. Many *Salmonella* serovars harbor P22-like prophages, suggesting prophage-mediated transmission of host DNA should not be underestimated^77^. In addition, due to alarming increase of drug resistance and their threat to human health, the World Health Organization has defined top priority pathogens, including *Acinetobacter*, *E. coli*, *Shigella*, and *Pseudomonas*^75^. These extremely recalcitrant pathogens are known to harbor high numbers of prophages, which may be induced and promote horizontal gene transfer of toxins and resistance genes. Paradoxically, it might be an advantage to treat bacterial infection in combination with an antioxidant to reduce the induction of the resident prophages. Glutathione detoxifies hydrogen peroxide to water, which indirectly lowers the formation of hydroxyl radicals and reduces the risk of DNA damage^42,78^. This could also reduce the mutagenic effect of fluoroquinolones or β-lactam antibiotics, as sub-antibiotic concentrations have been reported to promote mutations, and a linear correlation between ROS formation and mutation frequency has been observed, coinciding with the evolution of antibiotic resistance^79^. These results further suggest that the *lepA* mutant, which produces less ROS upon drug treatment and exhibits a reduced SOS response, would therefore accumulate fewer mutations and develop less drug resistance per cell. However, due to the increased survival of persister cells in this mutant, particularly after ciprofloxacin treatment, the reduction in ROS-mediated mutation rate could be compensated.

On the other hand, treatment of *S*. Typhimurium with ciprofloxacin and glutathione increased the subpopulation lacking prophage antirepressor/mCherry reporter induction and enhanced persister cell formation, presumably by reducing ROS-mediated DNA damage (Fig. 4). Nevertheless, a substantial subpopulation still showed antirepressor induction, as indicated by a strong mCherry signal. It is possible that the addition of glutathione partially protects prophage-related proteins from ROS-mediated damage in some cells, which could allow higher levels of prophage induction in this subpopulation. This might also contribute to the observation that glutathione had little apparent effect on plaque formation, as a subpopulation with strong prophage induction could mask the presence of cells without induction (Fig. 3G). It has been shown that *S*. Typhimurium forms persister cells in the host tissue when mice were treated with antibiotics^80^. After completion of the antibiotic regimen, the remaining persister cells migrated back into the gut lumen and transferred a plasmid encoding an extended-spectrum β-lactamase (ESPL), to susceptible *E. coli* recipients. Therefore, to determine an optimal treatment strategy to combat pathogens and to ensure complete eradication, further research is needed to clarify whether ROS-mediated killing could be used to our advantage or whether it will have negative effects on the spread of drug resistance.

## Conclusion

**Fig. 7:**
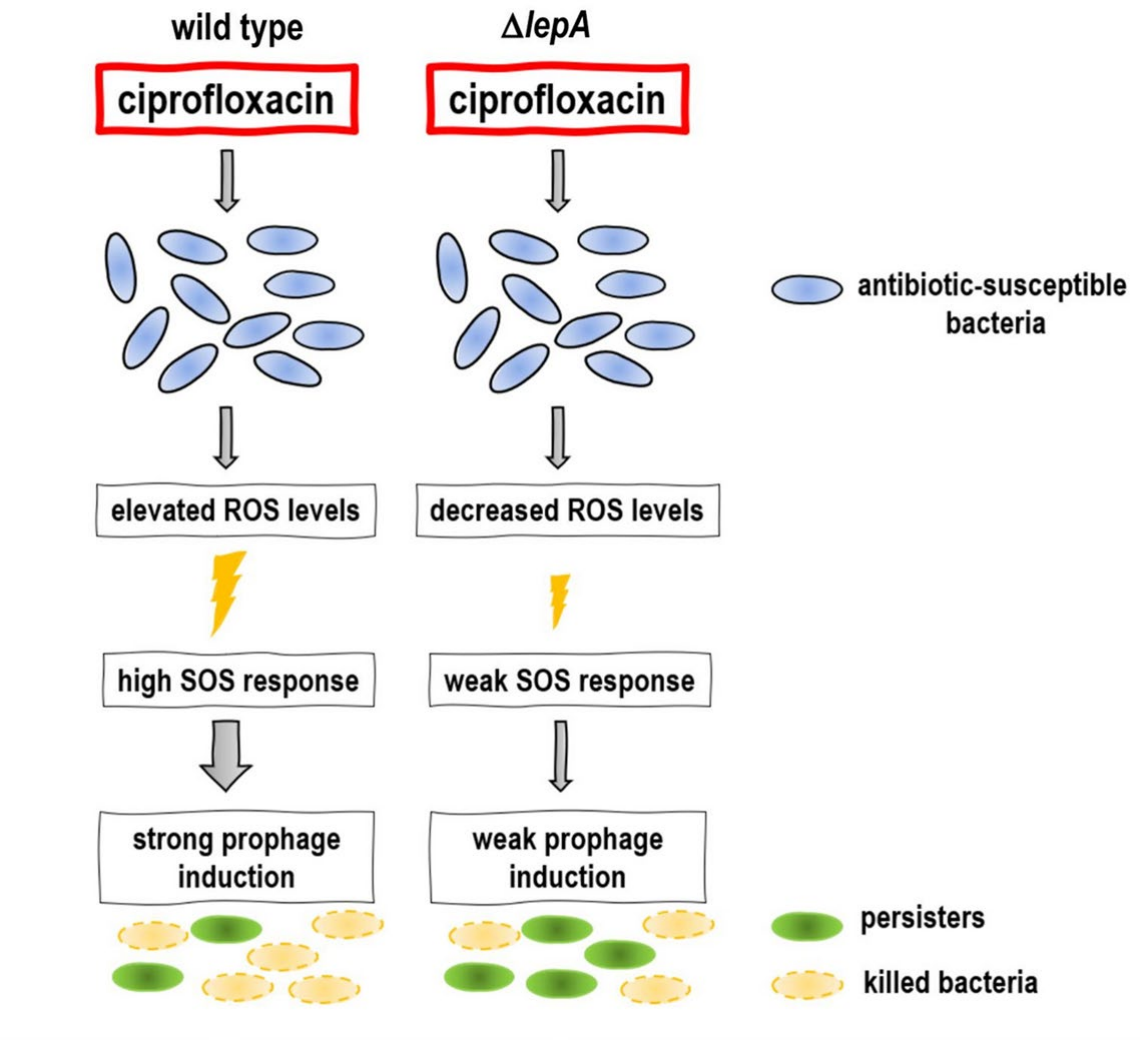
Proposed model of ROS- and prophage-mediated killing. Drug-susceptible wild type bacteria treated with ciprofloxacin exhibit increased formation of reactive oxygen species (ROS). The combination of DNA damage and elevated ROS levels results in a stronger SOS response, which in turn more efficiently activates prophages, leading to enhanced bacterial killing and a reduction in the subpopulation of drug-tolerant persister cells. In contrast to the Δ*lepA* mutant, which shows reduced ROS formation and reduced prophage induction.

Deletion of *lepA* in *S*. Typhimurium resulted in increased persister cell survival following ciprofloxacin treatment. This phenotype was associated with reduced ROS formation and decreased prophage induction, allowing a larger subpopulation of *lepA* mutants to survive antibiotic exposure (Fig. 7). These results indicate that ROS can enhance bacterial killing via prophage induction and, consequently, may increase the efficacy of antibacterial therapies. Furthermore, our findings provide new insight into the potential of temperate phage-antibiotic synergy as a strategy to improve the effectiveness of available antibiotics. Importantly, these results should be considered when interpreting apparently contradictory outcomes between studies, as prophages can significantly influence drug susceptibility, and prophage activation is affected by growth phase, activation pathways, nutrient availability, oxygen levels, and the concentration and class of the antibiotic used^16,81,82^.

## Materials and methods

### Media and antibiotics

All experiments were performed in lysogeny broth (LB Lennox) purchased from Carl Roth. The antibiotics were ciprofloxacin (Sigma-Aldrich), ampicillin (Carl Roth) and kanamycin (Carl Roth).

### Bacterial strains and plasmids

Strains and plasmids used in this study are listed in supplementary Tables S1 and S4 (Supplementary Material 1). The wild type background is *Salmonella enterica* subsp. *enterica* serovar Typhimurium (8640 also known as strain ATCC 14028s), as used in previous studies^14,15^. Gene deletions were introduced via P22 phage transduction using standard protocols, or via one-step inactivation (gene deletion) and replacement with a kanamycin-resistance cassette as described by Datsenko and Wanner^83^. Kanamycin-resistant colonies from P22 transductions were further purified on green plates to avoid carry-over of transducing P22 bacteriophage^84^. Successful gene deletions and transductions were confirmed by PCR. Elimination of the kanamycin resistance cassette was performed by transformation of clones with the FLP recombinase plasmid pCP20, followed by screening for loss of kanamycin-resistance, and subsequent elimination of pCP20 by growth at 37 °C in the absence of selection, as previously described^83,85^. Construction of the prophage-free strains has been previously described^16^.

To construct plasmid pSeb1, sequence encoding mCherry was amplified from plasmid pFCcGi^86^ using the primers indicated in supplementary Table S3 which introduce restriction sites for *Eco*RI and *Hin*dIII for cloning into the plasmid p2795^87^. Plasmid pSeb1 was subsequently used as a template for generation of a PCR product for gene deletion/replacement of target genes with mCherry following the protocol of Datsenko and Wanner (2000). All plasmids used in this study are listed in Table S4 (Supplementary Material 1).

### MIC determination

The minimum inhibitory concentration (MIC) determination was slightly modified from CLSI guidelines and performed as described in Braetz et al.^14^, using flat-bottomed 96-well plates (Corning). Briefly, wells containing a dilution series of the antibiotics were inoculated with 10^5^ bacteria, and the plates were incubated over-night at 37 °C under humid conditions to avoid evaporation of the medium (LB). The optical density (600 nm) was determined at time points t0 and 20 h later (t20) using a BioTek Synergy HT plate reader. The MIC for the antibiotics are as follows: ciprofloxacin 0.125 µg/ml (5 x MIC = 0.625 µg/ml; 32 x MIC = 4 µg/ml), ampicillin 0.78 µg/ml (5 x MIC = 3.9 µg/ml; 32 x MIC = 25 µg/ml), kanamycin 3.12 µg/ml (5 x MIC = 15.6 µg/ml) and chloramphenicol (5 x MIC = 6.25 µg/ml) (see Table S5).

### Persister assays/Time-killing assays

Glass culture tubes containing 6 ml of LB were inoculated with one colony from an LB plate which had been streaked out the day before and incubated over-night at 37 °C. Liquid cultures were incubated at 37 °C with aeration until they reached the mid-logarithmic phase (OD_600_ = 0.5). To determine persister cell formation, 6 ml of fresh LB was inoculated with 5 × 10^6^/ml bacteria from the mid-logarithmic phase culture followed by immediate removal of an aliquot for CFU determination (time point t0), and subsequent addition of antibiotics at 5x MIC or 32x MIC with continued shaking at 37 °C^16^. At the indicated time points, aliquots were removed, washed and plated for determinations of surviving CFU.

The resuscitation assay was performed according to Morishige et al. (2013)^40^. After four hours of treatment with 5x MIC ciprofloxacin, the bacteria were washed and resuspended in M9 medium supplemented with 50 mM pyruvate. Subsequently, the bacteria were incubated at 37 °C with shaking for one hour and plated on LB agar plates. The plates were incubated for at least 24 h for CFU determination.

### Detection of hydroxyl radicals

Hydroxyphenylfluorescein (HPF; ThermoFisher) was used to detect the formation of hydroxyl radicals after treatment with ciprofloxacin, ampicillin and kanamycin^15^. The bacteria were incubated to the mid-logarithmic phase as described above and subsequently transferred into 400 µl of fresh LB to achieve a CFU of 10^7^/ml bacteria. HPF was added at a final concentration of 100 µM together with the indicated antibiotic and incubated at 37 °C with aeration. For hydroxyl radical determination, an aliquot was removed, washed and the pellet resuspended in 1 x PBS. ROS levels (fluorescence) were determined for at least 100,000 events using a CytoFLEX flow cytometer (Beckman Coulter) with excitation at 488 nm and emission collected at 525/40 nm.

### Oxygen consumption

To measure oxygen consumption, we used OxoPlates (PreSens), which are 96-well round-bottom plates containing a polymer film with two different dyes at the bottom of each well, an indicator dye (I_Indicator_) and a reference dye (I_Reference_). The phosphorescence intensity of the indicator dye depends on the oxygen availability in the medium, whereas the reference dye is oxygen-independent. Oxygen consumption was determined by calculating the ratio of I_Indicator_ to I_Reference_ (I_R_ = I_Indicator_/I_Reference_), which corresponds to the oxygen partial pressure. Bacteria were grown to mid-logarithmic phase, diluted to 5 × 10⁶ cells/ml, and 200 µl per well were added to the OxoPlates in triplicate. Ciprofloxacin was added at concentrations of 5x MIC and 32x MIC. Each well was then covered with paraffin oil to prevent gas exchange, ensuring that only the oxygen consumption within the LB medium was measured. Fluorescence signals were recorded using a BioTek plate reader with excitation at 540 nm and emission at 650 nm (indicator dye) and 590 nm (reference dye).

### Real-Time PCR

The strains were incubated to the mid-logarithmic phase before treatment with the indicated antibiotic for two hours. Subsequently, the RNA was extracted using RNeasy Mini Kit from Qiagen according to the manufacturer´s protocols. The extracted RNA (400 ng) was reverse-transcribed into cDNA using reverse transcriptase (ThermoFisher), from which approximately 30 ng per well was used for the Real-Time PCR, as previously described in^10,16^. To detect the expression of the desired gene, Power™ SYBR™ Green Master Mix (ThermoFisher) was used with the corresponding primers listed in Table S2 (Supplementary Material 1). The housekeeping gene *trpA* was used for normalization^10,16,49^. Transcriptional activation in both strains was quantified, normalized to *trpA*, and the ratio between the wild type and the Δ*lepA* mutant was determined. Results are presented as relative induction for the indicated gene (mutant vs. wild type).

### SOS-dependent prophage induction

The strains 11326, SB283 and SB287 (Table 1 in Supplementary Material 1) were incubated as described above for the persister asssays in LB until reaching the exponential phase. To measure the SOS response-dependent induction of the Gifsy-1 prophage, the expression of a GfoA anti-repressor::mCherry transcriptional fusion was used^10^. The expression of *gfoA* is directly controlled by the bacterial host SOS response regulator, LexA^45^. Increased expression of GfoA is therefore an indicator of the SOS response and a prerequisite for prophage induction. The bacteria were exposed to 5 x MIC ciprofloxacin or ampicillin, respectively. After three hours of incubation at 37 °C with shaking (200 rpm), the bacteria were washed and resuspended in 1 x PBS. To determine the mCherry signal (Ex: 587 nm; Em: 610 nm), at least 200,000 events were measured using a CytoFLEX flow cytometer (Beckman Coulter).

### Plaque assay

We used MS1487 (wild type) and SB532 (Δ*lepA*), both carrying an inducible Gifsy-1 prophage. To ensure proper excision of Gifsy-1, we deleted *lepA* 123 base pairs downstream of the start codon, thereby preserving the excision recognition site of the prophage^88^. Bacteria were grown to mid-logarithmic phase and subsequently treated with 5x MIC/32x MIC ciprofloxacin for the indicated time. After centrifugation, the supernatant was transferred into a new Eppendorf tube, and 50 µl chloroform was added. The treated supernatant was then spotted onto soft agar containing the appropriate recipient strain (MS480)^89^.

### Confocal microscopy, image processing and analysis

The strains SB531 and SB501 carrying the katG-mScarlet-I construct were incubated as for the persister assay to mid-logarithmic phase in LB. Subsequently, 1 µl was transferred onto LB agarose pads (1.5% low melting agararose), which were held in place by silicone isolators A/A purchased from Electron Microscopy Sciences (size 13 mm dia x 0.1 mm depth). The agarose pads were covered using cover glasses from Zeiss (18 mm x 18 mm; 0.170 +/− 0.005 mm). Afterwards, the microscopic slides were incubated for two hours at 37 °C and then prepared for imaging. Confocal microscopy was performed at the Service Unit Microscopy of the Veterinary Centre for Resistance Research (TZR) with an inverted Leica Stellaris 8 FALCON system (Leica Microsystems), equipped with a 405 nm laser, a tunable white light laser (WLL, 440–790 nm), and Power HyD detectors. The microscope was operated by LAS X software version 4.8.1.29271. Images were acquired with a HC PL APO 63×/1.40 oil immersion objective. mScarlet-I was excited at 570 nm using the WLL set to 1.0% intensity, applying the unidirectional standard scanning mode with 4 line accumulations at 700 Hz scan speed. Fluorescence emission was collected between 575 and 650 nm using a HyD S detector in intensity mode (gain 2.5) and the pinhole set to one airy unit. A brightfield image was acquired simultaneously using a Trans-PMT detector. A zoom of 1.5 was applied resulting in a pixel size of 120 nm (@ 1024 × 1024 pixel) and image stacks consisting of 18 optical sections with a z-step size of 300 nm were acquired.

To quantify KatG-mScarlet intensity in individual bacteria, projections of images stacks were generated (sum projections of the KatG-mScarlet-I channel and extend depth of field (EDF) projections comprising 3–4 in-focus sections of the transmission channel) using Fiji/ImageJ^90^. Next, bacteria were segmented based on the projected transmission channel using Omnipose (version 1.0.7.dev20) with the provided pretrained ‘bact_phase_omni’ model^90^. Resulting mask images together with the sum projections of the KatG-mScarlet-I channel were loaded into CellProfiler (version 4.2.8) for further analysis. The used analysis pipeline is provided in Supplementary Material 3. In brief, after removing masks touching image borders, size and shape parameters of the remaining masks were measured and used to remove dirt particles incorrectly identified as bacteria. Next, the size and shape of all remaining objects as well as the intensity in the KatG-mScarlet-I channel was measured and exported for final data analysis. For quality control purposes, outlines of the bacteria were overlayed with the KatG-mScarlet-I images and saved.

Fiji/ImageJ was used to merge representative EDF projections of the brightfield channel with maximum intensity projections of the KatG-mScarlet-I channel and to linearly adjust brightness and contrast for display purposes. Intensity of the KatG-mScarlet-I channel was adjusted identical for all displayed images. Samples were randomized and blinded during image acquisition and analysis. The mScarlet-I signal was normalized to the respective untreated control and expressed as the fold change relative to the corresponding untreated strain.

### Lysis assay

The bacteria were incubated in LB to mid-logarithmic phase, diluted to 5 × 10⁶ cells/ml, and transferred Into 48-well plates. Ciprofloxacin and ampicillin were added at 5x MIC, and the OD₆₀₀ was measured at the indicated time points while shaking at 37 °C.

### Statistical analysis

Experiments were conducted in at least three independent biological replicates. Results are shown as mean ± standard deviation (SD). Statistical significance was evaluated using an unpaired two-tailed t test with Welch’s correction. Significance levels were defined as *p < 0.05, **p < 0.01 and ***p < 0.001.

## Supplementary Information

Below is the link to the electronic supplementary material.

Supplementary Material 1

Supplementary Material 2

Supplementary Material 3

## Data Availability

The raw data used in this study to calculate statistical significance are provided in the supplementary materials under the file labeled Supplementary Material 2.

## References

[1] Harms, A., Maisonneuve, E. & Gerdes, K. Mechanisms of bacterial persistence during stress and antibiotic exposure. *Science* **354**, aaf4268 (2016). 27980159 10.1126/science.aaf4268

[2] Maisonneuve, E. & Gerdes, K. Molecular mechanisms underlying bacterial persisters. *Cell* **157**, 539–548 (2014). 24766804 10.1016/j.cell.2014.02.050

[3] Moyed, H. S. & Bertrand, K. P. hipA, a newly recognized gene of Escherichia coli K-12 that affects frequency of persistence after inhibition of murein synthesis. *J. Bacteriol.* **155**, 768–775 (1983). 6348026 10.1128/jb.155.2.768-775.1983 PMC217749

[4] Theodore, A., Lewis, K. & Vulic, M. Tolerance of Escherichia coli to fluoroquinolone antibiotics depends on specific components of the SOS response pathway. *Genetics* **195**, 1265–1276 (2013). 24077306 10.1534/genetics.113.152306 PMC3832272

[5] Wu, Y., Vulic, M., Keren, I. & Lewis, K. Role of oxidative stress in persister tolerance. *Antimicrob. Agents Chemother.* **56**, 4922–4926 (2012). 22777047 10.1128/AAC.00921-12 PMC3421885

[6] Pontes, M. H. & Groisman, E. A. Slow growth determines nonheritable antibiotic resistance in Salmonella enterica. *Sci. Signal.* **12**, eaax3938 (2019). 31363068 10.1126/scisignal.aax3938 PMC7206539

[7] Cheverton, A. M. et al. A Salmonella Toxin Promotes Persister Formation through Acetylation of tRNA. *Mol. Cell.* **63**, 86–96 (2016). 27264868 10.1016/j.molcel.2016.05.002 PMC4942678

[8] Dorr, T., Vulic, M. & Lewis, K. Ciprofloxacin causes persister formation by inducing the TisB toxin in Escherichia coli. *PLoS Biol.* **8**, e1000317 (2010). 20186264 10.1371/journal.pbio.1000317 PMC2826370

[9] Nguyen, D. et al. Active starvation responses mediate antibiotic tolerance in biofilms and nutrient-limited bacteria. *Science* **334**, 982–986 (2011). 22096200 10.1126/science.1211037 PMC4046891

[10] Braetz, S. et al. TisB enables antibiotic tolerance in Salmonella by preventing prophage induction through ATP depletion. *PLoS Pathog*. **21**, e1013498 (2025). 40982487 10.1371/journal.ppat.1013498 PMC12626290

[11] Svenningsen, M. S., Veress, A., Harms, A., Mitarai, N. & Semsey, S. Birth and Resuscitation of (p)ppGpp Induced Antibiotic Tolerant Persister Cells. *Sci. Rep.* **9**, 6056 (2019). 30988388 10.1038/s41598-019-42403-7 PMC6465370

[12] Shan, Y. et al. ATP-dependent persister formation in Escherichia coli. *MBio* **8**, 10–1128 (2017). 10.1128/mBio.02267-16 PMC5296605 28174313

[13] Conlon, B. P. et al. Persister formation in Staphylococcus aureus is associated with ATP depletion. *Nat. Microbiol.* **1**, 16051 (2016). 27572649 10.1038/nmicrobiol.2016.51 PMC4932909

[14] Braetz, S., Schwerk, P., Thompson, A., Tedin, K. & Fulde, M. The role of ATP pools in persister cell formation in (fluoro)quinolone-susceptible and -resistant strains of Salmonella enterica ser. Typhimurium. *Vet. Microbiol.* **210**, 116–123 (2017). 29103680 10.1016/j.vetmic.2017.09.007

[15] Braetz, S., Schwerk, P., Thompson, A., Tedin, K. & Fulde, M. Salmonella Central Carbon Metabolism Enhances Bactericidal Killing by Fluoroquinolone Antibiotics. *Antimicrob. Agents Chemother.* **66**, e0234421 (2022). 35658490 10.1128/aac.02344-21 PMC9295562

[16] Braetz, S., Schwerk, P., Figueroa-Bossi, N., Tedin, K. & Fulde, M. Prophage Gifsy-1 Induction in Salmonella enterica Serovar Typhimurium Reduces Persister Cell Formation after Ciprofloxacin Exposure. *Microbiol. Spectr.*10.1128/spectrum.01874-23 (2023). e0187423. 37306609 10.1128/spectrum.01874-23 PMC10433948

[17] Mmolawa, P. T., Schmieger, H. & Heuzenroeder, M. W. Bacteriophage ST64B, a genetic mosaic of genes from diverse sources isolated from Salmonella enterica serovar typhimurium DT 64. *J. Bacteriol.* **185**, 6481–6485 (2003). 14563886 10.1128/JB.185.21.6481-6485.2003 PMC219385

[18] Figueroa-Bossi, N., Uzzau, S., Maloriol, D. & Bossi, L. Variable assortment of prophages provides a transferable repertoire of pathogenic determinants in Salmonella. *Mol. Microbiol.* **39**, 260–271 (2001). 11136448 10.1046/j.1365-2958.2001.02234.x

[19] Figueroa-Bossi, N., Coissac, E., Netter, P. & Bossi, L. Unsuspected prophage-like elements in Salmonella typhimurium. *Mol. Microbiol.* **25**, 161–173 (1997). 11902718 10.1046/j.1365-2958.1997.4451807.x

[20] Mulvey, M. R. et al. Ciprofloxacin-resistant Salmonella enterica serovar Kentucky in Canada. *Emerg. Infect. Dis.* **19**, 999–1001 (2013). 23735312 10.3201/eid1906.121351 PMC3713822

[21] Froshauer, S., Silvia, A. M., Chidambaram, M., Sharma, B. & Weinstock, G. M. Sensitization of bacteria to danofloxacin by temperate prophages. *Antimicrob. Agents Chemother.* **40**, 1561–1563 (1996). 8726041 10.1128/aac.40.6.1561 PMC163371

[22] Qin, Y. et al. The highly conserved LepA is a ribosomal elongation factor that back-translocates the ribosome. *Cell* **127**, 721–733 (2006). 17110332 10.1016/j.cell.2006.09.037

[23] Evans, R. N., Blaha, G., Bailey, S. & Steitz, T. A. The structure of LepA, the ribosomal back translocase. *Proc. Natl. Acad. Sci. U S A*. **105**, 4673–4678 (2008). 18362332 10.1073/pnas.0801308105 PMC2290774

[24] Gibbs, M. R. et al. Conserved GTPase LepA (Elongation Factor 4) functions in biogenesis of the 30S subunit of the 70S ribosome. *Proc. Natl. Acad. Sci. U S A*. **114**, 980–985 (2017). 28096346 10.1073/pnas.1613665114 PMC5293072

[25] Balakrishnan, R., Oman, K., Shoji, S., Bundschuh, R. & Fredrick, K. The conserved GTPase LepA contributes mainly to translation initiation in Escherichia coli. *Nucleic Acids Res.* **42**, 13370–13383 (2014). 25378333 10.1093/nar/gku1098 PMC4245954

[26] Shoji, S., Janssen, B. D., Hayes, C. S. & Fredrick, K. Translation factor LepA contributes to tellurite resistance in Escherichia coli but plays no apparent role in the fidelity of protein synthesis. *Biochimie* **92**, 157–163 (2010). 19925844 10.1016/j.biochi.2009.11.002 PMC2815024

[27] Bijlsma, J. J., Lie, A. L. M., Nootenboom, I. C., Vandenbroucke-Grauls, C. M. & Kusters, J. G. Identification of loci essential for the growth of Helicobacter pylori under acidic conditions. *J. Infect. Dis.* **182**, 1566–1569 (2000). 11023484 10.1086/315855

[28] Pech, M. et al. Elongation factor 4 (EF4/LepA) accelerates protein synthesis at increased Mg2 + concentrations. *Proc. Natl. Acad. Sci. U S A*. **108**, 3199–3203 (2011). 21300907 10.1073/pnas.1012994108 PMC3044372

[29] Dibb, N. J. Wolfe, lep operon proximal gene is not required for growth or secretion by Escherichia coli. *J. Bacteriol.* **166**, 83–87 (1986). 3514582 10.1128/jb.166.1.83-87.1986 PMC214560

[30] Badu-Nkansah, A. & Sello, J. K. Deletion of the elongation factor 4 gene (lepA) in Streptomyces coelicolor enhances the production of the calcium-dependent antibiotic. *FEMS Microbiol. Lett.* **311**, 147–151 (2010). 20735483 10.1111/j.1574-6968.2010.02083.x

[31] Wang, B. W., Zhu, J. H. & Javid, B. Clinically relevant mutations in mycobacterial LepA cause rifampicin-specific phenotypic resistance. *Sci. Rep.* **10**, 8402 (2020). 32439911 10.1038/s41598-020-65308-2 PMC7242378

[32] Fishbein, S. R. S. et al. The conserved translation factor LepA is required for optimal synthesis of a porin family in Mycobacterium smegmatis. *J. Bacteriol.* **203**, 10–128 (2020). 10.1128/JB.00604-20 PMC8095456 33361193

[33] Li, L. et al. Ribosomal elongation factor 4 promotes cell death associated with lethal stress. *mBio* **5**, e01708 (2014). 25491353 10.1128/mBio.01708-14 PMC4324249

[34] Kohanski, M. A., Dwyer, D. J., Hayete, B., Lawrence, C. A. & Collins, J. J. A common mechanism of cellular death induced by bactericidal antibiotics. *Cell* **130**, 797–810 (2007). 17803904 10.1016/j.cell.2007.06.049

[35] Dwyer, D. J. et al. Antibiotics induce redox-related physiological alterations as part of their lethality. *Proc. Natl. Acad. Sci. U S A*. **111**, E2100–2109 (2014). 24803433 10.1073/pnas.1401876111 PMC4034191

[36] Foti, J. J., Devadoss, B., Winkler, J. A., Collins, J. J. & Walker, G. C. Oxidation of the guanine nucleotide pool underlies cell death by bactericidal antibiotics. *Science* **336**, 315–319 (2012). 22517853 10.1126/science.1219192 PMC3357493

[37] Belenky, P. et al. Bactericidal Antibiotics Induce Toxic Metabolic Perturbations that Lead to Cellular Damage. *Cell. Rep.* **13**, 968–980 (2015). 26565910 10.1016/j.celrep.2015.09.059 PMC4648786

[38] Vilcheze, C. et al. Enhanced respiration prevents drug tolerance and drug resistance in Mycobacterium tuberculosis. *Proc. Natl. Acad. Sci. U S A*. **114**, 4495–4500 (2017). 28396391 10.1073/pnas.1704376114 PMC5410800

[39] Lobritz, M. A. et al. Antibiotic efficacy is linked to bacterial cellular respiration. *Proc. Natl. Acad. Sci. U S A*. **112**, 8173–8180 (2015). 26100898 10.1073/pnas.1509743112 PMC4500273

[40] Morishige, Y., Fujimori, K. & Amano, F. Differential resuscitative effect of pyruvate and its analogues on VBNC (viable but non-culturable) Salmonella. *Microbes Environ.* **28**, 180–186 (2013). 23595023 10.1264/jsme2.ME12174 PMC4070669

[41] Shirname-More, L., Rossman, T. G., Troll, W., Teebor, G. W. & Frenkel, K. Genetic effects of 5-hydroxymethyl-2’-deoxyuridine, a product of ionizing radiation. *Mutat. Res.* **178**, 177–186 (1987). 2953970 10.1016/0027-5107(87)90267-3

[42] Imlay, J. A., Chin, S. M. & Linn, S. Toxic DNA damage by hydrogen peroxide through the Fenton reaction in vivo and in vitro. *Science* **240**, 640–642 (1988). 2834821 10.1126/science.2834821

[43] Luan, G., Hong, Y., Drlica, K. & Zhao, X. Suppression of reactive oxygen species accumulation accounts for paradoxical bacterial survival at high quinolone concentration. *Antimicrob. Agents Chemother.* **62**, 10–128 (2018). 10.1128/AAC.01622-17 PMC5826153 29229642

[44] Keren, I., Wu, Y., Inocencio, J., Mulcahy, L. R. & Lewis, K. Killing by bactericidal antibiotics does not depend on reactive oxygen species. *Science* **339**, 1213–1216 (2013). 23471410 10.1126/science.1232688

[45] Lemire, S., Figueroa-Bossi, N. & Bossi, L. Bacteriophage crosstalk: coordination of prophage induction by trans-acting antirepressors. *PLoS Genet.* **7**, e1002149 (2011). 21731505 10.1371/journal.pgen.1002149 PMC3121763

[46] Alonso, A., Pucciarelli, M. G., Figueroa-Bossi, N., Garcia-del, F. & Portillo Increased excision of the Salmonella prophage ST64B caused by a deficiency in Dam methylase. *J. Bacteriol.* **187**, 7901–7911 (2005). 16291663 10.1128/JB.187.23.7901-7911.2005 PMC1291290

[47] Roca, A. I. & Cox, M. M. RecA protein: structure, function, and role in recombinational DNA repair. *Prog Nucleic Acid Res. Mol. Biol.* **56**, 129–223 (1997). 9187054 10.1016/s0079-6603(08)61005-3

[48] Turner, J. et al. The Chemical Relationship Among Beta-Lactam Antibiotics and Potential Impacts on Reactivity and Decomposition. *Front. Microbiol.* **13**, 807955 (2022). 35401470 10.3389/fmicb.2022.807955 PMC8988990

[49] Campoy, S. et al. Induction of the SOS response by bacteriophage lytic development in Salmonella enterica. *Virology* **351**, 360–367 (2006). 16713610 10.1016/j.virol.2006.04.001

[50] Hong, Y., Zeng, J., Wang, X., Drlica, K. & Zhao, X. Post-stress bacterial cell death mediated by reactive oxygen species. *Proc. Natl. Acad. Sci. U S A*. **116**, 10064–10071 (2019). 30948634 10.1073/pnas.1901730116 PMC6525477

[51] Trusca, D., Scott, S., Thompson, C. & Bramhill, D. Bacterial SOS checkpoint protein SulA inhibits polymerization of purified FtsZ cell division protein. *J. Bacteriol.* **180**, 3946–3953 (1998). 9683493 10.1128/jb.180.15.3946-3953.1998 PMC107380

[52] Mechler, L. et al. A novel point mutation promotes growth phase-dependent daptomycin tolerance in Staphylococcus aureus. *Antimicrob. Agents Chemother.* **59**, 5366–5376 (2015). 26100694 10.1128/AAC.00643-15 PMC4538524

[53] Levin-Reisman, I. et al. Antibiotic tolerance facilitates the evolution of resistance. *Science* **355**, 826–830 (2017). 28183996 10.1126/science.aaj2191

[54] Gutierrez, A. et al. Understanding and Sensitizing Density-Dependent Persistence to Quinolone Antibiotics. *Mol. Cell.* **68**, 1147–1154e1143 (2017). 29225037 10.1016/j.molcel.2017.11.012

[55] Woodmansee, A. N. & Imlay, J. A. Reduced flavins promote oxidative DNA damage in non-respiring Escherichia coli by delivering electrons to intracellular free iron. *J. Biol. Chem.* **277**, 34055–34066 (2002). 12080063 10.1074/jbc.M203977200

[56] Crumplin, G. C. & Smith, J. T. Nalidixic acid: an antibacterial paradox. *Antimicrob. Agents Chemother.* **8**, 251–261 (1975). 1101818 10.1128/aac.8.3.251 PMC429302

[57] Gao, Y. et al. Mammalian elongation factor 4 regulates mitochondrial translation essential for spermatogenesis. *Nat. Struct. Mol. Biol.* **23**, 441–449 (2016). 27065197 10.1038/nsmb.3206

[58] Bauerschmitt, H., Funes, S. & Herrmann, J. M. The membrane-bound GTPase Guf1 promotes mitochondrial protein synthesis under suboptimal conditions. *J. Biol. Chem.* **283**, 17139–17146 (2008). 18442968 10.1074/jbc.M710037200

[59] Barrett, T. C., Mok, W. W. K., Murawski, A. M. & Brynildsen, M. P. Enhanced antibiotic resistance development from fluoroquinolone persisters after a single exposure to antibiotic. *Nat. Commun.* **10**, 1177 (2019). 30862812 10.1038/s41467-019-09058-4 PMC6414640

[60] Fauvart, M., De Groote, V. N. & Michiels, J. Role of persister cells in chronic infections: clinical relevance and perspectives on anti-persister therapies. *J. Med. Microbiol.* **60**, 699–709 (2011). 21459912 10.1099/jmm.0.030932-0

[61] Paul, A., Nair, R. R., Jakkala, K., Pradhan, A. & Ajitkumar, P. Elevated Levels of Three Reactive Oxygen Species and Fe(II) in the Antibiotic-Surviving Population of Mycobacteria Facilitate De Novo Emergence of Genetic Resisters to Antibiotics. *Antimicrob. Agents Chemother.* **66**, e0228521 (2022). 35435709 10.1128/aac.02285-21 PMC9112956

[62] Bibb, L. A. & Hatfull, G. F. Integration and excision of the Mycobacterium tuberculosis prophage-like element, phiRv1. *Mol. Microbiol.* **45**, 1515–1526 (2002). 12354222 10.1046/j.1365-2958.2002.03130.x

[63] Strathdee, S. A., Hatfull, G. F., Mutalik, V. K. & Schooley, R. T. Phage therapy: From biological mechanisms to future directions. *Cell* **186**, 17–31 (2023). 36608652 10.1016/j.cell.2022.11.017 PMC9827498

[64] Al-Anany, A. M., Fatima, R. & Hynes, A. P. Temperate phage-antibiotic synergy eradicates bacteria through depletion of lysogens. *Cell. Rep.* **35**, 109172 (2021). 34038739 10.1016/j.celrep.2021.109172

[65] Eguchi, Y., Ogawa, T. & Ogawa, H. Cleavage of bacteriophage phi 80 CI repressor by RecA protein. *J. Mol. Biol.* **202**, 565–573 (1988). 3172227 10.1016/0022-2836(88)90286-0

[66] Galkin, V. E. et al. Cleavage of bacteriophage lambda cI repressor involves the RecA C-terminal domain. *J. Mol. Biol.* **385**, 779–787 (2009). 19013467 10.1016/j.jmb.2008.10.081 PMC2648975

[67] Harms, A., Fino, C., Sorensen, M. A. & Semsey, S. K. Gerdes prophages and growth dynamics confound experimental results with antibiotic-tolerant persister cells. *mBio* **8**, 10–128 (2017). 10.1128/mBio.01964-17 PMC5727415 29233898

[68] Maisonneuve, E., Castro-Camargo, M. & Gerdes, K. (p)ppGpp Controls Bacterial Persistence by Stochastic Induction of Toxin-Antitoxin Activity. *Cell* **172**, 1135 (2018). 29474913 10.1016/j.cell.2018.02.023

[69] Hoeksema, M. & Brul, S. B. H. Ter kuile, influence of reactive oxygen species on De Novo Acquisition of resistance to bactericidal antibiotics. *Antimicrob. Agents Chemother.* **62**, 10–128 (2018). 10.1128/AAC.02354-17 PMC5971590 29581120

[70] Glinkowska, M. et al. Influence of the Escherichia coli oxyR gene function on lambda prophage maintenance. *Arch. Microbiol.* **192**, 673–683 (2010). 20559623 10.1007/s00203-010-0596-2 PMC2903704

[71] Gu, X. et al. Bacterial Inactivation and Biofilm Disruption through Indigenous Prophage Activation Using Low-Intensity Cold Atmospheric Plasma. *Environ. Sci. Technol.* **56**, 8920–8931 (2022). 35438974 10.1021/acs.est.2c01516

[72] Zhu, W. et al. Involvement of OxyR and Dps in the repression of replication initiation by DsrA small RNA in Escherichia coli. *Gene* **882**, 147659 (2023). 37482259 10.1016/j.gene.2023.147659

[73] Kenzaka, T., Tani, K., Sakotani, A., Yamaguchi, N. & Nasu, M. High-frequency phage-mediated gene transfer among Escherichia coli cells, determined at the single-cell level. *Appl. Environ. Microbiol.* **73**, 3291–3299 (2007). 17384307 10.1128/AEM.02890-06 PMC1907122

[74] Schmieger, H. & Schicklmaier, P. Transduction of multiple drug resistance of Salmonella enterica serovar typhimurium DT104. *FEMS Microbiol. Lett.* **170**, 251–256 (1999). 9919675 10.1111/j.1574-6968.1999.tb13381.x

[75] Lopez-Leal, G. et al. Mining of thousands of prokaryotic genomes reveals high abundance of prophages with a strictly narrow host range. *mSystems* **7**, e0032622 (2022). 35880895 10.1128/msystems.00326-22 PMC9426530

[76] Busby, B., Kristensen, D. M. & Koonin, E. V. Contribution of phage-derived genomic islands to the virulence of facultative bacterial pathogens. *Environ. Microbiol.* **15**, 307–312 (2013). 23035931 10.1111/j.1462-2920.2012.02886.x PMC5866053

[77] Bearson, B. L. et al. The agricultural antibiotic carbadox induces phage-mediated gene transfer in Salmonella. *Front. Microbiol.* **5**, 52 (2014). 24575089 10.3389/fmicb.2014.00052 PMC3920066

[78] Goswami, M., Mangoli, S. H. & Jawali, N. Involvement of reactive oxygen species in the action of ciprofloxacin against Escherichia coli. *Antimicrob. Agents Chemother.* **50**, 949–954 (2006). 16495256 10.1128/AAC.50.3.949-954.2006 PMC1426460

[79] Kohanski, M. A., DePristo, M. A. & Collins, J. J. Sublethal antibiotic treatment leads to multidrug resistance via radical-induced mutagenesis. *Mol. Cell.* **37**, 311–320 (2010). 20159551 10.1016/j.molcel.2010.01.003 PMC2840266

[80] Bakkeren, E. et al. Salmonella persisters promote the spread of antibiotic resistance plasmids in the gut. *Nature* **573**, 276–280 (2019). 31485077 10.1038/s41586-019-1521-8 PMC6744281

[81] McDaniel, L. & Paul, J. H. Effect of nutrient addition and environmental factors on prophage induction in natural populations of marine synechococcus species. *Appl. Environ. Microbiol.* **71**, 842–850 (2005). 15691939 10.1128/AEM.71.2.842-850.2005 PMC546667

[82] Gavric, D. & Knezevic, P. Antibiotic-mediated prophage activation: Mechanisms, consequences, and therapeutic implications. *Int. J. Antimicrob. Agents*. **67**, 107655 (2026). 41167349 10.1016/j.ijantimicag.2025.107655

[83] Datsenko, K. A. & Wanner, B. L. One-step inactivation of chromosomal genes in Escherichia coli K-12 using PCR products. *Proc. Natl. Acad. Sci. U S A*. **97**, 6640–6645 (2000). 10829079 10.1073/pnas.120163297 PMC18686

[84] Chan, R. K., Botstein, D., Watanabe, T. & Ogata, Y. Specialized transduction of tetracycline resistance by phage P22 in Salmonella typhimurium. II. Properties of a high-frequency-transducing lysate. *Virology* **50**, 883–898 (1972). 4565618 10.1016/0042-6822(72)90442-4

[85] Cherepanov, P. P. & Wackernagel, W. Gene disruption in Escherichia coli: TcR and KmR cassettes with the option of Flp-catalyzed excision of the antibiotic-resistance determinant. *Gene* **158**, 9–14 (1995). 7789817 10.1016/0378-1119(95)00193-a

[86] Figueira, R., Watson, K. G., Holden, D. W. & Helaine, S. Identification of salmonella pathogenicity island-2 type III secretion system effectors involved in intramacrophage replication of S. enterica serovar typhimurium: implications for rational vaccine design. *mBio* **4**, e00065 (2013). 23592259 10.1128/mBio.00065-13 PMC3634603

[87] Husseiny, M. I. & Hensel, M. Rapid method for the construction of Salmonella enterica Serovar Typhimurium vaccine carrier strains. *Infect. Immun.* **73**, 1598–1605 (2005). 15731059 10.1128/IAI.73.3.1598-1605.2005 PMC1064926

[88] Waldor, M. K., Friedman, D. I. & Adhya, S. L. *Phages: their role in bacterial pathogenesis and biotechnology*ASM Press, Washington, D.C., pp. xvii, 450 p., 416 p. of plates. (2005).

[89] Sargen, M. R. & Helaine, S. A prophage competition element protects Salmonella from lysis. *Cell. Host Microbe*. **32**, 2063–2079 (2024). 39515326 10.1016/j.chom.2024.10.012 PMC11840918

[90] Cutler, K. J. et al. Omnipose: a high-precision morphology-independent solution for bacterial cell segmentation. *Nat. Methods*. **19**, 1438–1448 (2022). 36253643 10.1038/s41592-022-01639-4 PMC9636021

