# Supplementary material for: Reduced ROS-associated prophage induction in a lepA mutant contributes to increased fluoroquinolone persistence in Salmonella Typhimurium
Source: Sci Rep. 2026 Apr 17;16:12721. doi: 10.1038/s41598-026-47552-0 (PMC13090387; doi:10.1038/s41598-026-47552-0)
Supplement: Supplementary file 1 — Supplementary Material 1 [file 41598_2026_47552_MOESM1_ESM.pdf]

Supplementary data

**Table S1**: Strains used in this study		
Strain	Relevant Features*	Source
8640	*S.* Typhimurium ATCC 14028 Nal^R *gyrA*(D87Y)	(1)
10926	*S.* Typhimurium ATCC 14028 Nal^R ΔST64B	(2)
11126	*S.* Typhimurium ATCC 14028 Nal^R ΔGifsy-1 ΔGifsy--2 ΔGifsy-3 ΔST64B	(2)
11326	*S.* Typhimurium ATCC 14028 Nal^R ΔGifsy--2 ΔGifsy-3 ΔST64B	(2)
SB6	*S.* Typhimurium ATCC 14028 Nal^R Δ*lepA*	This study
SB283	*S.* Typhimurium ATCC 14028 Nal^R gfoA-dinI::mCherry ΔGifsy--2 ΔGifsy-3 ΔST64B Δ*lepA*	This study
SB287	*S.* Typhimurium ATCC 14028 Nal^R gfoA-dinI::mCherry ΔGifsy--2 ΔGifsy-3 ΔST64B	This study
SB421	*S.* Typhimurium ATCC 14028 Nal^R ΔGifsy-1 ΔGifsy--2 ΔGifsy-3 ΔST64B *lepA*::*kan*	This study
SB501	*S.* Typhimurium ATCC 14028 Nal^R katG-mScarlet-I Δ*lepA*	This study
10812	*S.* Typhimurium ATCC 14028 Nal^R *lexA3*(ind$^-$)	(3)
MS1487	*S.* Typhimurium ATCC 14028 ΔGifsy-2 ΔGifsy-3 ΔST64B	(4)
MS480	*S.* Typhimurium ATCC 14028 ΔGifsy-1 Gifsy-2 ΔGifsy-3 ΔST64B	(4)
SB531	*S.* Typhimurium ATCC 14028 Nal^R katG-mScarlet-I	This study
SB532	*S.* Typhimurium ATCC 14028 ΔGifsy-2 ΔGifsy-3 ΔST64B Δ+123lepA	This study
145	MG1655, *Escherichia coli*	(5)
SB561	MG1655 Δ*lepA*, *Escherichia coli*	This study
SB564	MG1655 Δ*lepA*, *Escherichia coli* + λ	This study
SB565	MG1655, *Escherichia coli* + λ	This study
*Abbreviations: Nal, nalidixic acid; *kan*, kanamycin resistance; *cat*, chloramphenicol resistance; +123, deletion of *lepA* starting 123 base pairs downstream of the start codon to enable excision of Gifsy-1.		

**Table S2**: Primers used in this study for qRT-PCR		
Primer	Sequence	Target
RecA-qPCR-For	GCCGATTTCGCCTTCGATTTCC	*recA*
RecA-qPCR-Rev	ATAACCTGCTCTGCTCTCAGCC	*recA*

**Table S3**: Primers used for generation of mutants and plasmids		
Primer	Sequence*	Target

k1	CAGTCATAGCCGAATAGCCT	*kan*(*aph*) gene (6)
k2	CGGTGCCCTGAATGAACTGC	*kan*(*aph*) gene (6)
kt	CGGCCACAGTCGATGAATCC	*kan*(*aph*) gene
LepA_For	GGAATGTCCACCGCTGTGTC	*lepA*
LepA_Rev	TGCCCGTCACCAGTGTGGCT	*lepA*
DinIF	GAAACAACATGCTAGCTTTTGC	*gfoA-dinI*
DinR	GTTGCGGGGATCAGAATTCCAG	*gfoA-dinI*
LepA_H1_P1	CATCAAGAAAAATTTATCAGCATAGCGAGTTGAAAAATTCATATTTATGTGTAG GCTGGAGCTGCTTCGA	*lepA*
LepA_H2_P2	CCAGAATCAGGGCAAACATGTTCGCCATGCCAACTCCTTAGGGATTATTTCTGC ATATGAATATCCTCCTTAG	*lepA*
lepAH1P1+123	ATCCAAATCTGCGGTGGCCTGTCTGACCGTGAAATGGAAGCTCAGGTACTTTG TGTAGGCTGGAGCTGCTTCGA	+123*lepA*
lepAH1P1/11126	CCTCTAAAGTCTGGTCAACCAGGCGTAAGGCATAATAATTTATCTCTATGTGTG TAGGCTGGAGCTGCTTCGA	*lepA*
DinI-GfoA-mCherry-F	TTGAATATTCTTTCTAACAGGTATACTGTGTTTATATACAGTAGTTAAATGTAG AGGGAATTATGGTGAGCAAGGGCGAGGAGGAT	*gfoA-dinI* (Gifsy-1)
DinI-GfoA-mCherry-R	CATGGTATTCTCCGTGGCGCAGAAGGTTAACGGTTGTTCAGGCCGTTGATTTC ATATTATCACGTGTAGGCTGGAGCTGCTTC	*gfoA-dinI* (Gifsy-1)
mCherry-EcoRI-For	CAGCTCGAGAATTCGAGATATACATATGGTGAGC	pFCcGi
mCherry-HindIII-Rev	AACTACCGCATTAAAGCTTTCGCGGCCGCT	pFCcGi
mCherry-For	GAGGGCGAGGGCGAGGGCCGC	pSeb1
mCherry-Rev	GTCCTGCAGGGAGGAGTCCTG	pSeb1

*Abbreviations: *kan*(*aph*), kanamycin resistance cassette of pKD4. Sequences for the common priming sites for amplification of the kanamycin resistance cassettes in pKD4 and internal kanamycin resistance gene (k1, k2, kt) are from Datsenko and Wanner (2000) (6).

Table S4: Plasmids used in this study as template for mutagenesis

plasmid	Relevant features	Source
pSeb1	mCherry + FRT-*aph*-FRT	(7)
pKD4	FRT-*kan*-FRT + *amp*	(6)
p2795	*aph* FRT in pSK; Amp^R Kan^R	(8)
pFCcGi	*rpsM::mCherry* and P_{BAD}*::gfpmut3a* promoter fusions in pFPV25.1 ($Carb^R$)	(9)
pCP20	FLP recombinas, Amp^R	(6)

Table S5: Minimum inhibitory concentration (MIC)

	**8640**	**SB6**	**11126**	**145**
antibiotics	MIC (µg/ml)			
ciprofloxacin	0.125	0.125	0.125	0.0039
ampicillin	0.78	0.78	N.D.	3.12

kanamycin	3.12	N.D	3.12	N.D.
chloramphenicol	1.25	N.D.	1.25	N.D.

*Abbreviations: N.D. = not determined.

References

1. S. Braetz, P. Schwerk, A. Thompson, K. Tedin, M. Fulde, The role of ATP pools in persister cell formation in (fluoro)quinolone-susceptible and -resistant strains of Salmonella enterica ser. Typhimurium. *Vet Microbiol* **210**, 116-123 (2017).
2. S. Braetz, P. Schwerk, N. Figueroa-Bossi, K. Tedin, M. Fulde, Prophage Gifsy-1 Induction in Salmonella enterica Serovar Typhimurium Reduces Persister Cell Formation after Ciprofloxacin Exposure. *Microbiol Spectr* 10.1128/spectrum.01874-23, e0187423 (2023).
3. K. Bunny, J. Liu, J. Roth, Phenotypes of lexA mutations in Salmonella enterica: evidence for a lethal lexA null phenotype due to the Fels-2 prophage. *J Bacteriol* **184**, 6235-6249 (2002).
4. M. R. Sargen, S. Helaine, A prophage competition element protects Salmonella from lysis. *Cell Host Microbe* **32**, 2063-2079 e2068 (2024).
5. F. R. Blattner *et al.*, The complete genome sequence of Escherichia coli K-12. *Science* **277**, 1453-1462 (1997).
6. K. A. Datsenko, B. L. Wanner, One-step inactivation of chromosomal genes in Escherichia coli K-12 using PCR products. *Proc Natl Acad Sci U S A* **97**, 6640-6645 (2000).
7. S. Braetz *et al.*, TisB enables antibiotic tolerance in Salmonella by preventing prophage induction through ATP depletion. *PLoS Pathog* **21**, e1013498 (2025).
8. M. I. Husseiny, M. Hensel, Rapid method for the construction of Salmonella enterica Serovar Typhimurium vaccine carrier strains. *Infect Immun* **73**, 1598-1605 (2005).
9. R. Figueira, K. G. Watson, D. W. Holden, S. Helaine, Identification of salmonella pathogenicity island-2 type III secretion system effectors involved in intramacrophage replication of S. enterica serovar typhimurium: implications for rational vaccine design. *mBio* **4**, e00065 (2013).

Supplementary figures

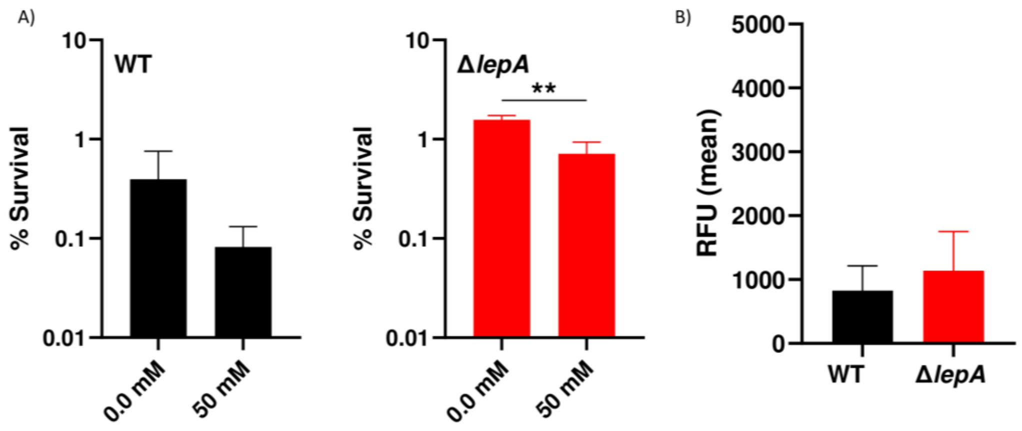

Supp Fig S1: Persister survival following pyruvate treatment and ROS formation in exponentially growing bacteria. A) The wild type (8640) and Δ*lepA* (SB6) mutant were treated with 5x MIC ciprofloxacin (0.625 µg/mL) for four hours, washed, and resuspended in M9 medium supplemented with 50 mM pyruvate prior to plating on LB agar plates. **B)** Determination of hydroxyl radical formation in both bacterial strains during exponential growth. At least three independent experiments were performed. Data are presented as mean ± standard deviation (SD). Statistical significance was assessed using an unpaired two-tailed t-test with Welch's correction. A p value < 0.05 was considered statistically significant (*p < 0.05; **p < 0.01).

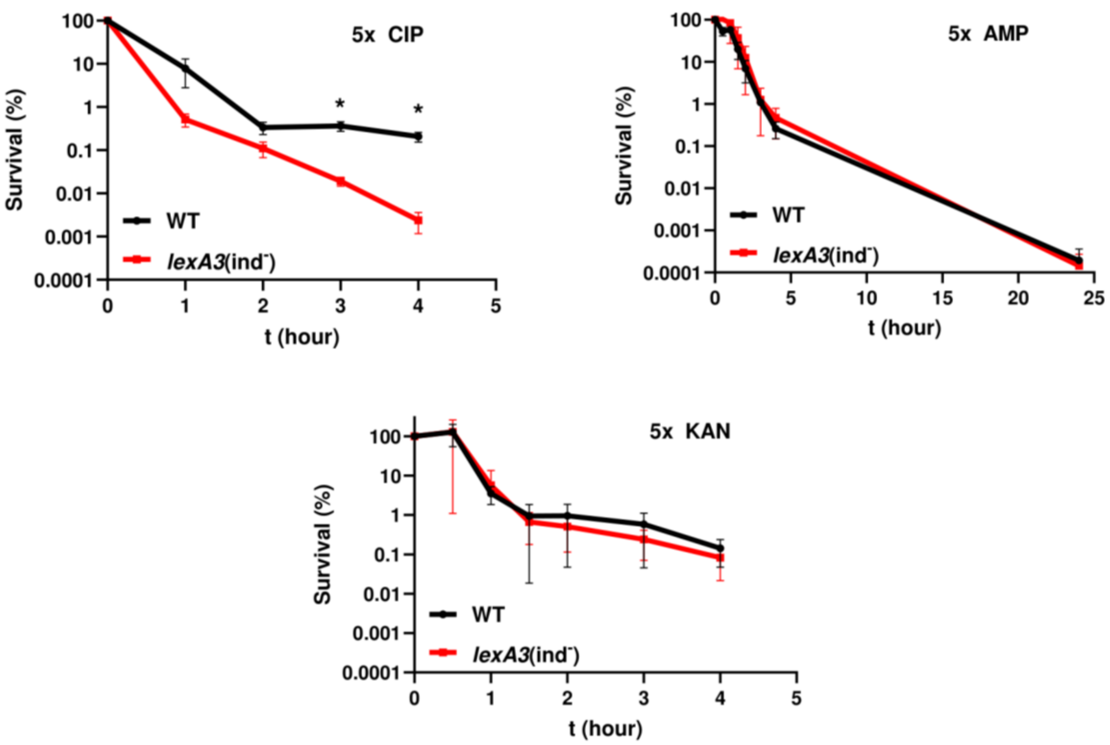

Supp Fig S2: Persister assays with a SOS response deficient LexA mutant (8640 = wild type; 10812 = *lexA3*(ind$^-$)). 5x MIC was used to treat exponentially growing bacteria. At least three independent experiments were performed. Data are presented as mean ± standard deviation (SD). Statistical significance was assessed using an unpaired two-tailed t-test with Welch's correction. A p value < 0.05 was considered statistically significant (*$p < 0.05$). (CIP = ciprofloxacin; AMP = ampicillin; KAN = kanamycin).

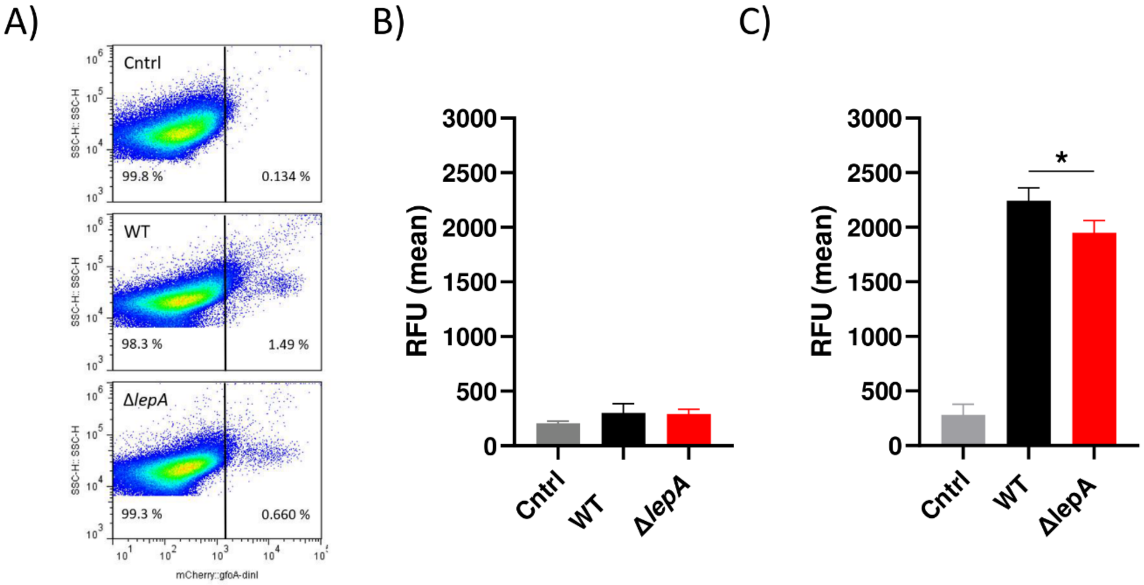

Supp Fig S3: FACS analysis of SB287 (WT mCherry::gfoA-dinI) and SB283 (Δ*lep*A mCherry::gfoA-dinI) after treatment with **A)** and **B)** 5 x MIC of ampicillin and **C)** 5 x MIC ciprofloxacin (for comparison). The solid line represents the gating strategy and Cntrl (control) stands for 11326, a strain without mCherry. At least three independent experiments were performed. Data are presented as mean ± standard deviation (SD). Statistical significance was assessed using an unpaired two-tailed t-test with Welch's correction. A p value < 0.05 was considered statistically significant (*p < 0.05).

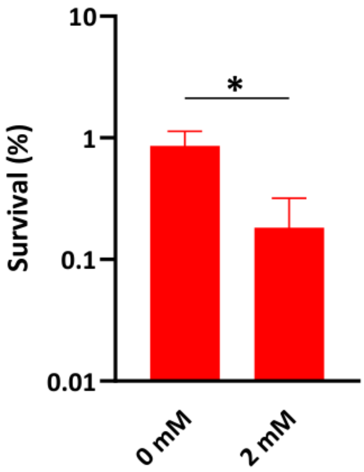

Supp Fig S4: Persister assay with the *lepA* mutant (SB6). Bacteria were treated with 5x MIC ciprofloxacin for four hours, either in the presence or absence of glutathione. At least three independent experiments were performed. Data are presented as mean ± standard deviation (SD). Statistical significance was assessed using an unpaired two-tailed t-test with Welch's correction. A p value < 0.05 was considered statistically significant (*p < 0.05).

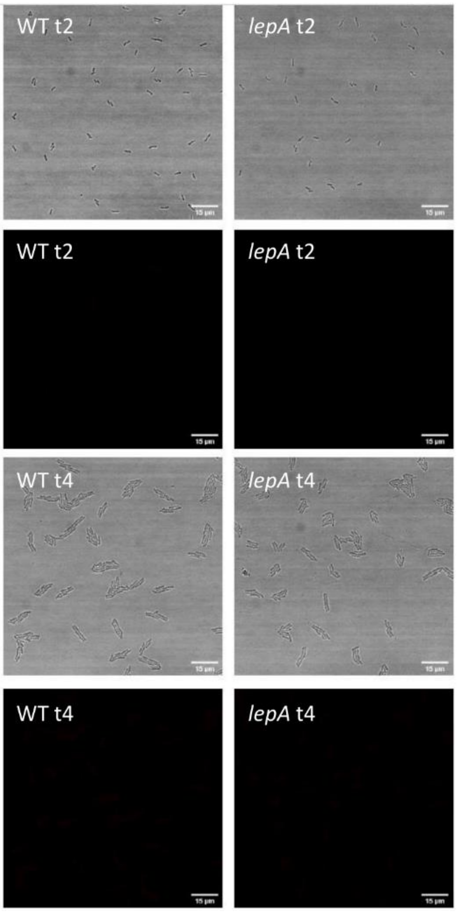

Supp Fig S5: Imaging of exponentially growing bacteria carrying a KatG–mScarlet-I fusion. t2 shows the wild type (SB531) and the Δ*lepA* mutant (SB501) after incubation for two hours in liquid LB and spotting on agarose pads. t4 indicates bacteria after two hours of growth on LB agarose pads. Bright-field and fluorescence images are shown.

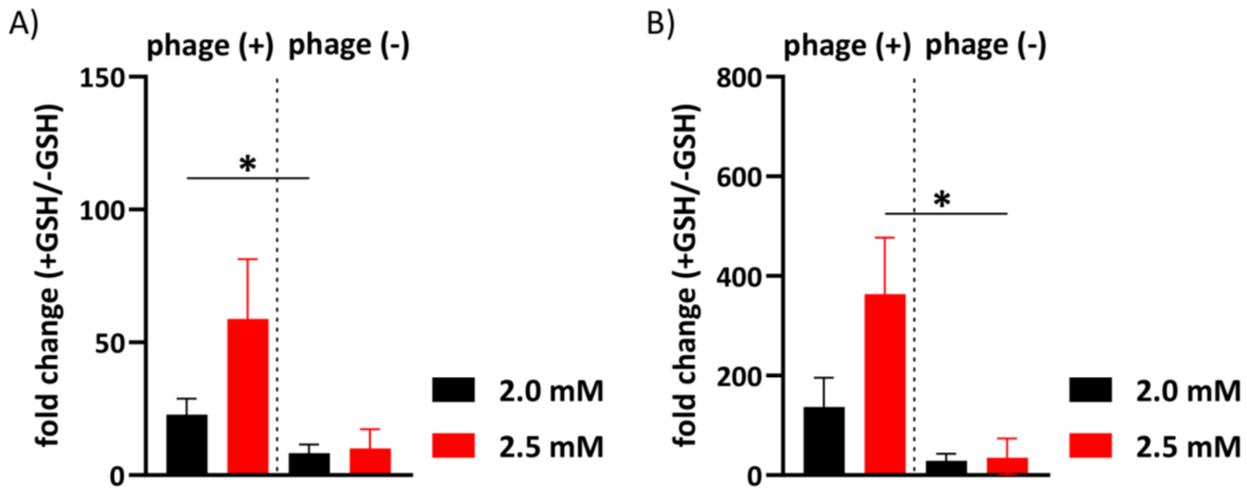

Supp Fig S6: Persister assays with *S*. Typhimurium (8640 = prophage positive strain) and the prophage-free variant (11126). **A)** Bacteria were either incubated for three hours with 32x MIC ciprofloxacin or **B)** for four hours. Survival was quantified, and the ratio between cultures without and with glutathione was calculated to assess the effect of glutathione on persister cell formation in each strain. At least three independent experiments were performed. Data are presented as mean ± standard deviation (SD). Statistical significance was assessed using an unpaired two-tailed t-test with Welch's correction. A p value < 0.05 was considered statistically significant (*$p < 0.05$; **$p < 0.01$).
