# Supplementary material for: Reduced ROS-associated prophage induction in a lepA mutant contributes to increased fluoroquinolone persistence in Salmonella Typhimurium
Source: Sci Rep. 2026 Apr 17;16:12721. doi: 10.1038/s41598-026-47552-0 (PMC13090387; doi:10.1038/s41598-026-47552-0)
Supplement: Supplementary file 3 — Supplementary Material 3 [file 41598_2026_47552_MOESM3_ESM.pdf]

```
CellProfiler Pipeline: http://www.cellprofiler.org
Version:5
DateRevision:428
GitHash:
ModuleCount:13
HasImagePlaneDetails:False

Images:[module_num:1|svn_version:'Unknown'|variable_revision_number:2|show_
window:False|notes:['To begin creating your project, use the Images module
to compile a list of files and/or folders that you want to analyze. You can
also specify a set of rules to include only the desired files in your
selected folders.']|batch_state:array([],
dtype=uint8)|enabled:True|wants_pause:False]
    :
    Filter images?:Images only
    Select the rule criteria:and (extension does isimage) (directory
doesnot containregexp "[\\\\/]\\.")

Metadata:[module_num:2|svn_version:'Unknown'|variable_revision_number:6|sho
w_window:False|notes:['The Metadata module optionally allows you to extract
information describing your images (i.e, metadata) which will be stored
along with your measurements. This information can be contained in the file
name and/or location, or in an external file.']|batch_state:array([],
dtype=uint8)|enabled:True|wants_pause:False]
    Extract metadata?:Yes
    Metadata data type:Text
    Metadata types:{}
    Extraction method count:2
    Metadata extraction method:Extract from file/folder names
    Metadata source:File name
    Regular expression to extract from file name:(?P<Sample>Sample_[0-
9]{1})_(?P<Position>[0-9]_(?P<Timepoint>[0-9]h))
    Regular expression to extract from folder name:(?P<Masks>masks)$
    Extract metadata from:All images
    Select the filtering criteria:and (file does contain "")
    Metadata file location:Elsewhere...|
    Match file and image metadata:[]
    Use case insensitive matching?:No
    Metadata file name:None
    Does cached metadata exist?:No
    Metadata extraction method:Extract from image file headers
    Metadata source:File name
    Regular expression to extract from file
name:^(?P<Plate>.*)_(?P<Well>[A-P][0-9]{2})_s(?P<Site>[0-
9])_w(?P<ChannelNumber>[0-9])
    Regular expression to extract from folder name:(?P<Date>[0-9]{4}_[0-
9]{2}_[0-9]{2})$
    Extract metadata from:All images
    Select the filtering criteria:and (file does contain "")
    Metadata file location:Elsewhere...|
    Match file and image metadata:[]
    Use case insensitive matching?:No
    Metadata file name:None
    Does cached metadata exist?:Yes

NamesAndTypes:[module_num:3|svn_version:'Unknown'|variable_revision_number:
8|show_window:False|notes:['The NamesAndTypes module allows you to assign a
meaningful name to each image by which other modules will refer to
it.']|batch_state:array([], dtype=uint8)|enabled:True|wants_pause:False]
    Assign a name to:Images matching rules
    Select the image type:Grayscale image
    Name to assign these images:DNA
```

```
    Match metadata:[{'mScarlet': 'Sample', 'Masks': 'Sample'}, {'mScarlet': 'FileLocation', 'Masks': 'FileLocation'}]
    Image set matching method:Metadata
    Set intensity range from:Image metadata
    Assignments count:2
    Single images count:0
    Maximum intensity:255.0
    Process as 3D?:No
    Relative pixel spacing in X:0.1803762
    Relative pixel spacing in Y:0.1803762
    Relative pixel spacing in Z:1.0
    Select the rule criteria:and (metadata does C "0")
    Name to assign these images:mScarlet
    Name to assign these objects:Cell
    Select the image type:Grayscale image
    Set intensity range from:Image metadata
    Maximum intensity:255.0
    Select the rule criteria:and (metadata does C "1")
    Name to assign these images:DNA
    Name to assign these objects:Masks
    Select the image type:Objects
    Set intensity range from:Image metadata
    Maximum intensity:255.0

Groups:[module_num:4|svn_version:'Unknown'|variable_revision_number:2|show_window:False|notes:['The Groups module optionally allows you to split your list of images into image subsets (groups) which will be processed independently of each other. Examples of groupings include screening batches, microtiter plates, time-lapse movies, etc.']|batch_state:array([], dtype=uint8)|enabled:True|wants_pause:False]
    Do you want to group your images?:Yes
    grouping metadata count:3
    Metadata category:FileLocation
    Metadata category:Position
    Metadata category:Timepoint

FilterObjects:[module_num:5|svn_version:'Unknown'|variable_revision_number:10|show_window:False|notes:[]|batch_state:array([], dtype=uint8)|enabled:True|wants_pause:False]
    Select the objects to filter:Masks
    Name the output objects:FilterBorderMasks
    Select the filtering mode:Image or mask border
    Select the filtering method:Limits
    Select the objects that contain the filtered objects:None
    Select the location of the rules or classifier file:Elsewhere...|
    Rules or classifier file name:rules.txt
    Class number:1
    Measurement count:1
    Additional object count:0
    Assign overlapping child to:Both parents
    Keep removed objects as a separate set?:No
    Name the objects removed by the filter:RemovedObjects
    Select the measurement to filter by:AreaShape_Area
    Filter using a minimum measurement value?:Yes
    Minimum value:0.0
    Filter using a maximum measurement value?:Yes
    Maximum value:1.0
    Allow fuzzy feature matching?:No

MeasureObjectSizeShape:[module_num:6|svn_version:'Unknown'|variable_revision_number:3|show_window:False|notes:[]|batch_state:array([], dtype=uint8)|enabled:True|wants_pause:False]
    Select object sets to measure:FilterBorderMasks
```

```
    Calculate the Zernike features?:Yes
    Calculate the advanced features?:No

FilterObjects:[module_num:7|svn_version:'Unknown'|variable_revision_number:
10|show_window:False|notes:[]|batch_state:array([],
dtype=uint8)|enabled:True|wants_pause:False]
    Select the objects to filter:FilterBorderMasks
    Name the output objects:FilterObjects
    Select the filtering mode:Measurements
    Select the filtering method:Limits
    Select the objects that contain the filtered objects:None
    Select the location of the rules or classifier file:Elsewhere...|
    Rules or classifier file name:rules.txt
    Class number:1
    Measurement count:3
    Additional object count:0
    Assign overlapping child to:Both parents
    Keep removed objects as a separate set?:No
    Name the objects removed by the filter:RemovedObjects
    Select the measurement to filter by:AreaShape_Area
    Filter using a minimum measurement value?:Yes
    Minimum value:150
    Filter using a maximum measurement value?:No
    Maximum value:1.0
    Select the measurement to filter by:AreaShape_MinorAxisLength
    Filter using a minimum measurement value?:Yes
    Minimum value:10.0
    Filter using a maximum measurement value?:No
    Maximum value:1.0
    Select the measurement to filter by:AreaShape_Eccentricity
    Filter using a minimum measurement value?:Yes
    Minimum value:0.90
    Filter using a maximum measurement value?:No
    Maximum value:1.0
    Allow fuzzy feature matching?:No

MeasureObjectIntensity:[module_num:8|svn_version:'Unknown'|variable_revisio
n_number:4|show_window:False|notes:[]|batch_state:array([],
dtype=uint8)|enabled:True|wants_pause:False]
    Select images to measure:mScarlet
    Select objects to measure:FilterObjects

MeasureObjectSizeShape:[module_num:9|svn_version:'Unknown'|variable_revisio
n_number:3|show_window:False|notes:[]|batch_state:array([],
dtype=uint8)|enabled:True|wants_pause:False]
    Select object sets to measure:FilterObjects
    Calculate the Zernike features?:Yes
    Calculate the advanced features?:No

RescaleIntensity:[module_num:10|svn_version:'Unknown'|variable_revision_num
ber:3|show_window:False|notes:[]|batch_state:array([],
dtype=uint8)|enabled:True|wants_pause:False]
    Select the input image:mScarlet
    Name the output image:RescaleIntensitymScarlet
    Rescaling method:Stretch each image to use the full intensity range
    Method to calculate the minimum intensity:Custom
    Method to calculate the maximum intensity:Custom
    Lower intensity limit for the input image:0.0
    Upper intensity limit for the input image:1.0
    Intensity range for the input image:0.0,1.0
    Intensity range for the output image:0.0,1.0
    Select image to match in maximum intensity:None
    Divisor value:1.0
```

```
    Divisor measurement:None

OverlayOutlines:[module_num:11|svn_version:'Unknown'|variable_revision_numb
er:4|show_window:False|notes:[]|batch_state:array([],
dtype=uint8)|enabled:True|wants_pause:False]
    Display outlines on a blank image?:No
    Select image on which to display outlines:RescaleIntensitymScarlet
    Name the output image:OverlayOutlinesBacteria
    Outline display mode:Color
    Select method to determine brightness of outlines:Max of image
    How to outline:Inner
    Select outline color:Red
    Select objects to display:FilterObjects

SaveImages:[module_num:12|svn_version:'Unknown'|variable_revision_number:16
|show_window:False|notes:[]|batch_state:array([],
dtype=uint8)|enabled:True|wants_pause:False]
    Select the type of image to save:Image
    Select the image to save:OverlayOutlinesBacteria
    Select method for constructing file names:From image filename
    Select image name for file prefix:mScarlet
    Enter single file name:OrigBlue
    Number of digits:4
    Append a suffix to the image file name?:Yes
    Text to append to the image name:_Outlines
    Saved file format:tiff
    Output file location:Elsewhere...|
    Image bit depth:8-bit integer
    Overwrite existing files without warning?:Yes
    When to save:Every cycle
    Record the file and path information to the saved image?:No
    Create subfolders in the output folder?:No
    Base image folder:Elsewhere...|
    How to save the series:T (Time)
    Save with lossless compression?:Yes

ExportToSpreadsheet:[module_num:13|svn_version:'Unknown'|variable_revision_
number:13|show_window:True|notes:[]|batch_state:array([],
dtype=uint8)|enabled:True|wants_pause:False]
    Select the column delimiter:Comma (",")
    Add image metadata columns to your object data file?:Yes
    Add image file and folder names to your object data file?:No
    Select the measurements to export:Yes
    Calculate the per-image mean values for object measurements?:No
    Calculate the per-image median values for object measurements?:No
    Calculate the per-image standard deviation values for object
measurements?:No
    Output file location:Elsewhere...|
    Create a GenePattern GCT file?:No
    Select source of sample row name:Metadata
    Select the image to use as the identifier:None
    Select the metadata to use as the identifier:None
    Export all measurement types?:No
    Press button to select
measurements:FilterObjects|AreaShape_Area,FilterObjects|Intensity_MaxIntens
ity_mScarlet,FilterObjects|Intensity_MinIntensityEdge_mScarlet,FilterObject
s|Intensity_MedianIntensity_mScarlet,FilterObjects|Intensity_IntegratedInte
nsity_mScarlet,FilterObjects|Intensity_MaxIntensityEdge_mScarlet,FilterObje
cts|Intensity_MeanIntensity_mScarlet,FilterObjects|Intensity_MassDisplaceme
nt_mScarlet,FilterObjects|Intensity_StdIntensity_mScarlet,FilterObjects|Int
ensity_UpperQuartileIntensity_mScarlet,FilterObjects|Intensity_MinIntensity
_mScarlet,FilterObjects|Intensity_MeanIntensityEdge_mScarlet,FilterObjects|
Intensity_MADIntensity_mScarlet,FilterObjects|Intensity_StdIntensityEdge_mS
```

```
carlet,FilterObjects|Intensity_IntegratedIntensityEdge_mScarlet,FilterObjec
ts|Intensity_LowerQuartileIntensity_mScarlet
    Representation of Nan/Inf:NaN
    Add a prefix to file names?:Yes
    Filename prefix:SingleCellAnalysis_
    Overwrite existing files without warning?:Yes
    Data to export:FilterObjects
    Combine these object measurements with those of the previous object?:No
    File name:DATA.csv
    Use the object name for the file name?:Yes
```
